# Supplementary material for: Neighbourhood environments for a healthy lifestyle among young single-person households experiencing housing poverty in Seoul, South Korea: a spatiotemporal qualitative study protocol
Source: BMJ Open. 2024 Jan 30;14(1):e077234. doi: 10.1136/bmjopen-2023-077234 (PMC10828882; doi:10.1136/bmjopen-2023-077234)
Supplement: Supplementary data [file bmjopen-2023-077234supp001.pdf]

Supplement Material 1. Interview questions

Activities	Interview questions
Prepare a timetable (Confirmation by displaying on the timetable based on participant responses)	**1. We'd like to draw a timeline of your daily life. Describe a typical day in your life.**<br>- How long has this lifestyle been going on?<br>- Is there a reason for this change in lifestyle (if it's different before or after COVID-19)?<br><br>**2. We'd like to ask your thoughts on "healthy living".**<br>- What do you think "healthy living" is about practicing or enjoying in your daily life?<br>- As you reflect on your day, describe any activities you maintain or try to do to stay healthy.<br>- If there's something you'd like to try to do for your health and you're not able to, please explain what you can't do and why.<br>- Thinking about your day, describe any activities that you do that contribute to poor health.<br>- Describe any reasons why you engage in the activity despite its health risks.
Prepare a neighborhood map<br>(Confirm by displaying on a map based on participant responses)	**3. We would like to ask you about places, spaces that you associate with 'healthy living'.**<br>- Looking at the map, please explain where you do/would like to do each of the activities related to "healthy living" that you mentioned earlier.<br>- Where is this space/place located? Please mark it on the map.<br>- What activities do you typically do in that space/location?<br>- Why did you choose that space/location, if any?<br>- What are the challenges of utilizing that space/location?<br>(*Need to check: the environment of your current home)
	**4. We'd like to ask you about your neighborhood's health-related environment.**<br>- Fill in the blanks in the sentence "One thing about my neighborhood, [ ], affects my health" and be specific about why.<br>- Show me on a map where you think my neighborhood exemplifies the characteristics you just described.<br>- Plot the route from your home to the location on a map.<br>- If you've been to or used these places, please describe what you

	did there, for what purpose, and what it was like.<br>- If there's one thing you'd like to see improved in your neighborhood for healthy living, please describe it.<br>- Assuming you live in the neighborhood you live in now for a long time to come, how do you think the meaning of your neighborhood or your use of it will change?<br>- Based on what we've talked about so far, plot on a map where you think your "neighborhood" is and where it ends.

Supplement Material 2. Checklist of field observation (Lambton Public Health, 2017)

Check areas	Check points
**HEALTHY Transportation Networks**	HAVE YOU...<br>☐ Supported a variety of transportation modes that are convenient and safe for all ages?<br>☐ Encouraged retail and commercial spaces that support active transportation with connections to cycling paths, trails, sidewalks or transit, if applicable?<br>☐ Required or encouraged homes and places of employment to be within a comfortable walking or biking distance of frequent destinations such as schools, parks, shops, and transit stops, if applicable?<br>☐ Designed roadways and access to buildings and services as pedestrian friendly
**HEALTHY Food Systems**	HAVE YOU...<br>☐ Supported and provided space to grow food (agriculture or neighborhood gardens)?<br>☐ Supported access to healthy and local food?<br>☐ Supported the integration of all of the pieces of the food system in your municipality to enhance the environmental, economic, social, and nutritional health of the community (Producing, Processing, Distributing, Accessing Food, Eating, and Managing Waste)?
**HEALTHY Neighborhood Design**	HAVE YOU...<br>☐ Encouraged mixed land-use designations in your community?<br>☐ Adequately distributed trails, parks or recreation facilities in your community?<br>☐ Promoted or designed your community where people can easily connect with each other and a variety of day-to-day services?<br>☐ Encouraged the Three Cs of healthy neighborhoods: Complete, Compact and Connected?<br>☐ Examined hazard, safety and vandalism concerns in your community
**HEALTHY Natural Environments**	HAVE YOU...<br>☐ Made parks and natural areas of your community accessible to all demographics, safe and connected?

	☐ Protected and enhanced natural areas from development?<br>☐ Incorporated trees and natural features into all new and re-developments, including road work?<br>☐ Incorporated trees and natural features to reduce UV exposure, air pollution, water pollution and urban heat islands?
**HEALTHY Housing**	HAVE YOU...<br>☐ Promoted affordable housing programs?<br>☐ Regulated and enforced housing quality standards?<br>☐ Addressed any high frequencies of tenant/resident relocation or turnover?<br>☐ Designated housing within close proximity to other services and away from environmental hazards?

Supplement Material 3. Round-shaped timetables

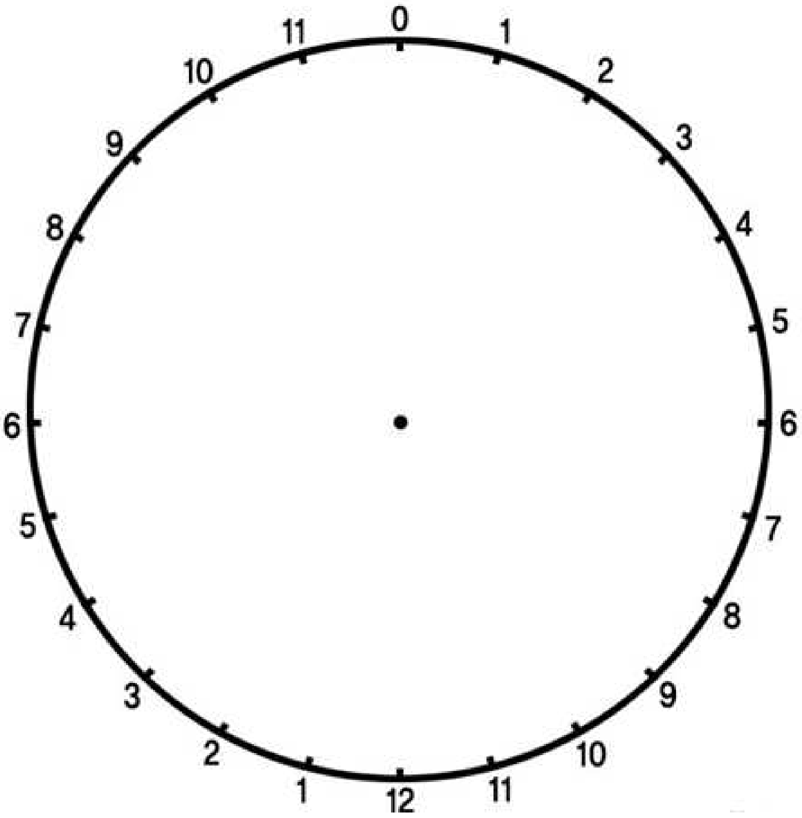

Supplement Material 4. Standards for reporting qualitative research (SRQR) checklist

No.	Topic	Items	Page/line no(s).
**Title and Abstract**			
S1	Title	Concise description of the nature and topic of the study Identifying the study as qualitative or indicating the approach (e.g., ethnography, grounded theory) or data collection methods (e.g., interview, focus group) is recommended	Page 1. L 1-2
S2	Abstract	Summary of key elements of the study using the abstract format of the intended publication; typically includes background, purpose, methods, results, and conclusions	Page 1. L4-28
**Introduction**			
S3	Problem formulation	Description and significance of the problem/phenomenon studied; review of relevant theory and empirical work; problem statement	Page 2. L41-85
S4	Purpose or research question	Purpose of the study and specific objectives or questions	Page 4. L86-96
**Methods**			
S5	Qualitative approach and research paradigm	Qualitative approach (e.g., ethnography, grounded theory, case study, phenomenology, narrative research) and guiding theory if appropriate; identifying the research paradigm (e.g., postpositivist, constructivist/ interpretivist) is also recommended; rationale	Page 4. L99-115
S6	Researcher characteristics and reflexivity	Researchers' characteristics that may influence the research, including personal attributes, qualifications/experience, relationship with participants, assumptions, and/or presuppositions; potential or actual interaction between researchers' characteristics and the research questions, approach, methods, results, and/or transferability	Page 9. L238-243
S7	Context	Setting/site and salient contextual factors; rationale	Page 5. L117-128
S8	Sampling strategy	How and why research participants, documents, or events were selected; criteria for deciding when no further sampling was necessary (e.g., sampling saturation); rationale	Page 5. L130-147
S9	Ethical issues pertaining to human subjects	Documentation of approval by an appropriate ethics review board and participant consent, or explanation for lack thereof; other confidentiality and data security issues	Page 10. L.263-274
S10	Data collection method	Types of data collected; details of data collection procedures including (as appropriate) start and stop dates of data collection and analysis, iterative process, triangulation of sources/methods, and modification of procedures in response to evolving study findings; rationale	Page 6. L148-212
S11	Data collection instruments and technologies	Description of instruments (e.g., interview guides, questionnaires) and devices (e.g., audio recorders) used for data collection; if/how the instrument(s) changed over the course of the study	Page 7. L165-167<br>Page 7. L180-184<br>Page 8. L196-202<br>Page 8. L204-207

S12	Units of study	Number and relevant characteristics of participants, documents, or events included in the study; level of participation (could be reported in results)	Page 5. L131-137
S13	Data processing	Methods for processing data prior to and during analysis, including transcription, data entry, data management and security, verification of data integrity, data coding, and anonymization/de-identification of excerpts	Page 7. L174-176<br>Page 9. L228-232
S14	Data analysis	Process by which inferences, themes, etc., were identified and developed, including the researchers involved in data analysis; usually references a specific paradigm or approach; rationale	Page 9. L213-237
S15	Techniques to enhance trustworthiness	Techniques to enhance trustworthiness and credibility of data analysis (e.g., member checking, audit trail, triangulation); rationale	Page 10. L244-252
**Results/findings**			
S16	Synthesis and interpretation	Main findings (e.g., interpretations, inferences, and themes); might include development of a theory or model, or integration with prior research or theory	N/A*
S17	Links to empirical data	Evidence (e.g., quotes, field notes, text excerpts, photographs) to substantiate analytic findings	N/A*
**Discussion**			
S18	Integration with prior work, implications, transferability, and contribution(s) to the field	Short summary of main findings; explanation of how findings and conclusions connect to, support, elaborate on, or challenge conclusions of earlier scholarship; discussion of scope of application/generalizability; identification of unique contribution(s) to scholarship in a discipline or field	N/A*
S19	Limitations	Trustworthiness and limitations of findings	Page 2. L29-39
**Others**			
S20	Conflict of interest	Potential sources of influence or perceived influence on study conduct and conclusions; how these were managed	Page 11. L281
S21	Funding	Sources of funding and other support; role of funders in data collection, interpretation, and reporting	Page 11. L279-280

N/A: These items do not apply to this manuscript, which is a research protocol.
