# Supplementary material for: Neighbourhood environments for a healthy lifestyle among young single-person households experiencing housing poverty in Seoul, South Korea: a spatiotemporal qualitative study protocol
Source: BMJ Open. 2024 Jan 30;14(1):e077234. doi: 10.1136/bmjopen-2023-077234 (PMC10828882; doi:10.1136/bmjopen-2023-077234)
Supplement: Author's manuscript [file bmjopen-2023-077234.draft_revisions.pdf]

BMJ Open

BMJ Open is committed to open peer review. As part of this commitment we make the peer review history of every article we publish publicly available.

When an article is published we post the peer reviewers' comments and the authors' responses online. We also post the versions of the paper that were used during peer review. These are the versions that the peer review comments apply to.

The versions of the paper that follow are the versions that were submitted during the peer review process. They are not the versions of record or the final published versions. They should not be cited or distributed as the published version of this manuscript.

BMJ Open is an open access journal and the full, final, typeset and author-corrected version of record of the manuscript is available on our site with no access controls, subscription charges or pay-per-view fees (http://bmjopen.bmj.com).

If you have any questions on BMJ Open's open peer review process please email info.bmjopen@bmj.com

BMJ Open

Neighborhood environments for a healthy lifestyle among young single-person households experiencing housing poverty in Seoul, South Korea: A spatiotemporal qualitative study protocol

Journal:	*BMJ Open*
Manuscript ID	bmjopen-2023-077234
Article Type:	Protocol
Date Submitted by the Author:	29-Jun-2023
Complete List of Authors:	Kim, Dong Ha; Seoul National University, Institute of Health and Environment<br>Lee, Jihyun; Seoul National University, Department of Public Health Sciences, Graduate School of Public Health<br>Yoo, Seunghyun; Seoul National University, Department of Public Health Sciences, Graduate School of Public Health; Seoul National University, Institute of Health and Environment
Keywords:	Health Equity, PUBLIC HEALTH, QUALITATIVE RESEARCH, Quality of Life

SCHOLARONE™
Manuscripts

Neighborhood environments for a healthy lifestyle among young single-person households experiencing housing poverty in Seoul, South Korea: A spatiotemporal qualitative study protocol

ABSTRACT

Introduction

The number of single-person households is increasing globally—including in South Korea, where single-person households account for over 30% of all households. Young single-person households in South Korea face health and housing problems. The neighborhood environment plays a significant role in maintaining a healthy lifestyle. To comprehensively understand the relationship between the neighborhood environment and a healthy lifestyle, it is crucial to collect data on the neighborhood environment and the perceived and objective aspects. This study aims to explore and describe the meaning, roles, and spatiotemporal characteristics of neighborhood environments for a healthy lifestyle in young single-person households experiencing housing poverty in Seoul, South Korea.

Methods and analysis

This ongoing study uses an extended qualitative GIS approach to explore a district in Seoul, that has the highest population density of young single-person households experiencing housing poverty. The study sample comprises young single-person households between the ages of 19 and 39 years who are experiencing housing poverty in the study area. Online and offline recruitment strategies are employed to ensure the inclusion of diverse perspectives. A multimethod approach is used that combines descriptive and spatiotemporal data collection techniques, such as individual in-depth interviews using cognitive maps, round-shaped timetables, field observations, and mobile GPS tracking. The data analysis encompasses thematic and content analyses to understand the neighborhood environment's perceived attributes and spatiotemporal characteristics of healthy lifestyles. In the integrated analysis, we plan to combine qualitative findings with living space and daily-life patterns using qualitative software and a hybrid relational database.

Ethics and dissemination

The Institutional Review Board approved the research protocol on May 18, 2021. The findings will be shared

For peer review only - http://bmjopen.bmj.com/site/about/guidelines.xhtml

at international conferences and published in academic journals. Additionally, an online seminar will share the results with policymakers, researchers, community organizations, and health workers working with young single-person households experiencing housing poverty.

Strengths and limitations of this study

- This study provides valuable insights by exploring the lived experiences of young single-person households living in housing poverty.
- This study helps to identify the meanings and values attached to young single-person households experiencing housing poverty in various places and to understand how these meanings are formed and interpreted.
- This study can be time-consuming and labor-intensive because it involves collecting and analyzing data from multiple sources.
- This study requires a high level of technical expertise to collect, manage, and analyze data, which can be a barrier for researchers unskilled in GIS technology and qualitative research methods.

BACKGROUND

Single-person households are becoming increasingly common worldwide. In European Union member states, the proportion of single-person households increased from 31% in 2010 to 34% in 2017 (1). According to the United States Census Bureau (2), the proportion of single-person households in the United States increased from 16.7% in 1969 to 28.4% in 2019, and the rate of increase is expected to accelerate over the next decade. Similar to most developed countries, South Korea (hereafter Korea) has experienced remarkable household fragmentation in recent years, leading to a trend toward individualized life courses. In 2000, single-person households, which accounted for only 15.5% of all households in Korea, began to account for the highest household composition ratio (27.2%), beating multi-person households in 2015. Subsequently, this figure rose to 33.4% by 2021 (3).

Compared to the international situation, young single-person households in Korea are at risk of facing health and housing problems. According to the Korean National Health and Nutrition Examination Survey (4) conducted in 2020, 63.2% of young single-person households aged 19–29 were either obese or overweight; 27.7% showed

For peer review only - http://bmjopen.bmj.com/site/about/guidelines.xhtml

symptoms of depression, and 29.3% were smokers. These figures exceed the average of Organization for Economic Co-operation and Development (OECD) member countries for the same period (5). The Korea Research Institute for Human Settlements (6) reported that 60% of young single-person households in Korea would spend more than 30% of their monthly income on housing rents in 2021. Additionally, according to the 2020 Korea Housing Survey (7), 9.6% of single-person households living in houses with less than 14 m^2 of residential space experienced difficulties in soundproofing, ventilation, heating, and kitchen facilities. These housing conditions are imperfect compared to the international minimum housing standards that consider detailed facilities and layouts such as kitchens, bathrooms, bathing facilities, privacy protection, appropriate hot water supply temperatures, and 25 m^2 per person (8). This suggests that young single-person households in Korea may not have sufficient resources within their homes to manage and protect their health adequately. The extent to compensate for and fulfill this shortfall within their neighborhood environment may be an important factor in determining whether they can maintain a healthy lifestyle.

Neighborhood environment refers to the physical, social, economic, and cultural factors that surround and influence residents within a specific geographic area (9). These factors include land use patterns, street safety, public transportation systems, living facilities, parks and green spaces, housing quality, and social and cultural opportunities. Additionally, the neighborhood environment has perceived and objective aspects (10). The *perceived aspect* of a neighborhood environment refers to how individuals perceive and experience their neighborhood (11). The *objective aspect* of the neighborhood environment, however, refers to the physical and structural characteristics of the neighborhood that can be measured quantitatively (11). The perceived and objective aspects of the neighborhood environment are significant in shaping individuals' experiences and health outcomes. Perceived aspects influence how individuals behave and engage with their communities, whereas objective aspects affect access to resources and services, safety, and overall well-being (12). By understanding the perceived and objective aspects of the neighborhood environment, policymakers, researchers, and community members can work together to create healthier and more livable neighborhoods.

Collecting data on various aspects such as time, space, and experience is crucial for comprehensively understanding the perceived and objective neighborhood environments related to healthy lifestyles. Time availability in neighborhood environments can form a pattern of participation in health behaviors (13), and spatial access to neighborhood environments can affect an individual's ability to maintain a healthy lifestyle (14).

For peer review only - http://bmjopen.bmj.com/site/about/guidelines.xhtml

Perceptions of neighborhood environments can also affect intentions, practices, and attitudes toward health behaviors (15). Therefore, considering these aspects in data collection can provide a more comprehensive understanding of the relationship between the neighborhood environment and healthy lifestyle of young single-person households experiencing housing poverty.

This study aims to explore and describe the meaning, roles, and spatiotemporal characteristics of neighborhood environments for a healthy lifestyle in young single-person households experiencing housing poverty in Seoul, Korea. The specific research questions are as follows:

1) What perspectives do young single-person households experiencing housing poverty have on their homes and neighborhoods in promoting a healthy lifestyle?

2) How do young single-person households experiencing housing poverty maintain or attempt to pursue a healthy lifestyle in their neighborhoods?

3) What attributes and characteristics of neighborhood environments are necessary to support healthy lifestyles in young, single-person households experiencing housing poverty?

4) What is the context and scope of perceived neighborhood boundaries for young single-person households experiencing housing poverty?

METHODS AND ANALYSIS

Study design

This study employs an extended qualitative GIS (QGIS) research design by adding time-use data from daily life. The original QGIS methodology integrates qualitative and geospatial methods to investigate the rich contextual details of spatial experiences in various formats (16). By merging qualitative methods with GIS-based techniques, QGIS provides an analytical framework for narrative inquiries into spatial experiences that have previously been unfeasible (17, 18). The advantage of visualizing textual information on a map, such as perceptions, attitudes, emotions, beliefs, and behaviors, is the ability to explore spatial and environmental contexts that may not be fully elucidated through interviews alone (19). By incorporating time aspects into these benefits, we expect to explore the daily time usage patterns of young single-person households experiencing housing poverty in their neighborhoods.

For peer review only - http://bmjopen.bmj.com/site/about/guidelines.xhtml

A schematic diagram of the extended qualitative GIS used in this study is shown in Figure 1. This diagram demonstrates how qualitative, spatial, and temporal data can be combined to produce a rich contextual understanding of people's lived experiences in their neighborhoods.

[Figure 1]

Setting

We selected an administrative district in Seoul with the highest density of young single-person households experiencing housing poverty. Our study is currently being conducted in Gwanak-gu, a southern district of Seoul (Figure 2). The district has three administrative neighborhoods with a total area of 29.6 km^2, of which 60% is occupied by mountainous terrain (20). As of September 2021, there were 164,000 single-person households in the district, making it the region with the largest number of single-person households in Korea (20). The main types of houses in the district are multiple complex houses with semi-basements, accounting for 55% of the total houses (20). The average per capita housing area was 28.2 m^2 (approximately 303 ft^2), and the monthly rent for a studio apartment was approximately 400,000 won (approximately 299.00 USD). Gwanak-gu is known as one of the districts with poor and low-cost residential environments (20). In addition, the district has a low-income community ("Go-si-chon" in Korean) where densely populated unemployed young people who prepare for the civil service examinations live (20).

[Figure 2]

Study sample and recruitment

Three administrative neighborhoods in Gwanak-gu where young single-person households are concentrated are included in our research as study areas. We are currently in the planning phase of conducting purposive sampling for single-person households between the ages of 19 and 39 experiencing housing poverty within the study areas. In this study, housing poverty is defined as residing in housing that either 1) fails to meet the Minimum Housing Standards in Korea, or 2) requires more than 30% of the monthly household income (21). The exclusion criteria for potential study participants include individuals who have lived in the study areas for less than six months and those who do not possess electronic and digital devices capable of conducting online interviews. Online and offline recruitment strategies are currently being employed to ensure the inclusion of diverse

For peer review only - http://bmjopen.bmj.com/site/about/guidelines.xhtml

perspectives from participants of various genders, ages, employment statuses, housing types, and residence periods. Online recruitment involves posting recruitment documents on social network service (SNS) platforms commonly used by young people in the study areas with the cooperation of online community managers. Offline recruitment entails attaching recruitment documents to telephone poles, bulletin boards, and walls located in areas frequented by local young people as well as visiting public service centers that rent spaces for local young people to recruit participants. Participants are being recruited until data saturation is achieved, when new information no longer produces significant changes to the codebook (22). The saturation point of study participants is expected to be approximately 9–17 people per neighborhood, based on previous research (23), and with additional participants to confirm saturation, the number of study participants is expected to be approximately 55.

Data collection

We are currently employing a multi-method approach to gather descriptive and spatiotemporal information on the neighborhood environment for healthy lifestyles among young single-person households experiencing housing poverty. This approach combines descriptive data collection techniques, such as individual in-depth interviews; spatial data collection techniques, such as cognitive mapping, field observations, and mobile GPS tracking; and temporal data collection techniques, such as drawing round-shaped timetables (e.g., hourly schedules represented in a circular format). The data collection process involves both non-human research (field observations) and human subject research. We are conducting human subject research in the order of individual in-depth interviews using cognitive maps and round-shaped timetables, followed by mobile GPS tracking (Figure 3). Data collection has been ongoing since September 2022.

[Figure 3]

Descriptive data collection

Individual in-depth interviews are an effective and proactive qualitative research method that can be used to understand the lives and experiences of research participants in their language and perspective and to discover their inner world (24). Different types of individual in-depth interviews can be conducted depending on the means, number of research participants, and interview format. The most well-known types include open, semi-structured, and structured interviews, in openness to interview questions. For this ongoing study, we are conducting semi-

For peer review only - http://bmjopen.bmj.com/site/about/guidelines.xhtml

structured interviews with young single-person households experiencing housing poverty. The interviews are being conducted face-to-face and through the online videoconferencing platform Zoom, depending on the participants' preferences. The researchers explain the study and obtain consent from the participants regarding their participation in addition to recording and use of their interviews, which will only be transcribed and used for analysis. Participants are informed that they can stop the interview at any time if they wished, and the informed consent form makes it clear that participation is entirely voluntary.

After obtaining consent, we conduct a survey to collect demographic information, including sex, age, occupation, length of residence, housing characteristics, and health status. Following the survey, we conduct one-on-one in-depth interviews using semi-structured questions to gain insights into the perceived attributes of neighborhood environments related to healthy lifestyles (Supplement material 1). We record and transcribe the interviews into electronic documents with an average turnaround time of five days. The expected average interview time per participant is approximately one hour and thirty minutes.

Spatial data collection

Field observation is a spatial research method in which a researcher observes phenomena related to a research topic while moving around a research site (25). Currently, we are following a procedure for conducting field observations. We use Google Maps and Street View to examine the neighborhood environment and plan observation paths, schedules, and boundaries. To investigate the physical characteristics of the neighborhood environment and the contents that change depending on the observation time, we use The Healthy Community Checklist (26) . We are manually recording the collected information on field observation sheets (Supplement material 2), which will be digitized for future reference and organized by date and neighborhood.

Techniques to visually represent an individual's or group's mental model of the physical environment are known as cognitive mapping (27). This involves asking participants to either map from their memories or annotate existing maps with information that reflects their perceptions, knowledge, and experiences of the environment. In this study, the researchers conduct activities during one-on-one interviews with the participants to create cognitive maps. Participants are asked to mark directly on the map by recalling places and experiences related to healthy living. Upon completion of the cognitive map, the researchers ask the participants to explain the main activities in the marked places, their reason for selecting the place, and any difficulties they encountered while using the place. Finally, the participants are asked to indicate the environmental characteristics of their neighborhood that

For peer review only - http://bmjopen.bmj.com/site/about/guidelines.xhtml

affect their health, with the perceived boundaries of their neighborhood. We expect cognitive maps to provide insights into how people navigate and use their environments, perceive and prioritize different features and landmarks, and conceptualize their spatial relationships.

Mobile GPS can easily track the spatial movement of participants' daily activity radius in real-time (28). In this study, the participants wear the Garmin Forerunner device and record their daily lives during the survey period. The mobile GPS devices help confirm the range of the participants' walking activity radii and identify their movement paths and locations where they stayed in the neighborhood. The participants' data, including distance traveled, duration of movement, movement path, and place of residence, are automatically synchronized with Google Maps and stored daily. The research team conducts the tracking period once for three days, including weekdays (two days) and weekends (one day), based on previous research evidence (29).

Temporal data collection

During the one-on-one in-depth interviews, we use a technique that creates a round-shaped timetable to understand the participants' 24-hour daily routines, separated into weekdays and weekends. We divide a 24-hour day into equal segments and assign activities or events to each segment based on start and end times. Each segment is labeled and represented by a circle, similar to a clock face (Supplement material 3). We ask the participants to complete a daily schedule based on weekday and weekend routines. Once completed, the researchers review the participants' schedules and ask for additional explanations of their daily neighborhood activities. Finally, we ask the participants to describe activities related to a healthy lifestyle and explain why, when, and how often they engaged in them. We expect the round timetable to outline how individuals allocate their time, the duration and frequency of different activities, and the temporal patterns of their behavior.

Data analysis and integration

We will conduct six stages of thematic analysis (30) for the in-depth interview data. In the first stage, we will develop a general framework for the participants' perceptions of the neighborhood environment related to a healthy lifestyle by repeatedly reading the interview data. In the second stage, we will conduct an open coding process to identify all meaning units related to the perception of the neighborhood environment that affects participants' healthy lifestyles, such as meaning, attributes, and roles. In the third stage, we will compare and

For peer review only - http://bmjopen.bmj.com/site/about/guidelines.xhtml

contrast the relationships between the generated codes to identify categories. In the fourth stage, we will review the tentatively derived themes to determine whether they match the research purpose and reflect the data content. In the fifth stage, we will refine the derived themes into precise definitions through a recursive process of returning data for comparative analysis. Based on the final analysis results, we will describe in detail and present appropriate explanations and discussions with the research purpose.

We plan to perform content analysis using spatiotemporal data. Content analysis offers a systematic and objective approach to draw accurate inferences from verbal, visual, or written data, enabling the description and quantification of specific phenomena (31) . We will extract information such as time usage patterns (amount of time and time of day), activity types, and activity range (within/outside the neighborhood) from the timetables created by individual study participants and analyzing them through overlapping analysis. We will convert the cognitive map data provided by the participants and the field observation data surveyed by the researchers into a digital map using the Quantum GIS program to quantitatively analyze spatial characteristics (location, density, radius, distance, and distribution). We will analyze the mobile log data collected through mobile GPS tracking for neighborhood mobility patterns using the statistical software R and Google Maps.

During the integrated analysis phase, we plan to combine qualitative results with living space and daily life pattern results using Atlas.ti qualitative software and a hybrid relational database. The hybrid relational database will allow us to create a descriptive data database under the attribute table of spatiotemporal data, which can integrate various types of qualitative data, such as images, texts, and videos, into spatial data (32). Atlas.ti can process text data and non-text multimedia data and can be linked to GIS and codes (33).

ETHICS AND DISSEMINATION

The Institutional Review Board of the researchers' affiliated institution approved the research on May 18, 2021 (IRB No. 2106/002-018). Before participating in the study, participants are asked to provide written informed consent to ensure complete anonymity and confidentiality. The informed consent form explains the purpose of the recording, possible risks and discomfort, possible benefits, confidentiality of information, and withdrawal procedures in detail. Participants' anonymity is protected, and no identifiable characteristics are specified in the recorded conversations.

We will disseminate the findings to the academic society by presenting them at international conferences on

For peer review only - http://bmjopen.bmj.com/site/about/guidelines.xhtml

urban health and publishing them in academic journals. In addition, we plan to conduct an online seminar/webinar to share and discuss our research findings with policymakers, researchers, community organizations, and health workers who plan and implement policies and programs for young single-person households experiencing housing poverty.

Contributors D.H.K., J.L., and S.Y. conceived the study. D.H.K., J.L., and S.Y. contributed to the development of the study design and final protocols for sample selection and data collection. D.H.K. and J.L. contributed to writing the manuscript. S.Y. reviewed and approved the final version of the paper.

Funding This work was supported by a National Research Foundation (NRF) grant funded by the Korean government (Ministry of Science, ICT, and Future Planning; NRF- 2020R1A2C2012463).

Competing interests None declared.

Patient consent for publication Not required.

Provenance and peer review Not commissioned; externally peer reviewed

For peer review only - http://bmjopen.bmj.com/site/about/guidelines.xhtml

REFERENCES

1 Eurostat. Eurostat Regional Yearbook: 2019 Edition. Eurostat Publications Office 2019. doi: 10.2785/1522

2 United States Census Bureau. One-person households on the rise [online]. 2019. https://www.census.gov/library/visualizations/2019/comm/one-person-households.html (accessed 7 April 2023).

3 Statistics Korea. Single-Person Households in 2021 Statistics. Statistics Korea 2021.

4 Ministry of Health and Welfare, Korea Disease Control and Prevention Agency. Announcement of the Results of the 8th National Health and Nutrition Survey for the 2nd Year. 2020.

5 Organization for Economic Co-operation and Development. Health at a Glance 2021: OECD Indicators. OECD Publishing. 2021. doi: 10.1787/ae3016b9-en

6 Park M, Woo J. Complementary Strategies for Housing Vulnerability by Age Group of Single-Person Households. Sejong: Korea Research Institute for Human Settlements 2021:16–18.

7 Korea Research Institute for Human Settlements. 2020 Housing Survey. Sejong: Ministry of Land, Infrastructure and Transport 2020:91‒92.

8 Yoo D, Lee J, Jang E. An international comparative study to shift from minimum to decent housing standard. JAIK 2022;38:47‒58.

9 Ding D, Sallis JF, Kerr J, et al. Neighborhood environment and physical activity among youth a review. Am J Prev Med 2011;41:442–55. doi: 10.1016/j.amepre.2011.06.036.

10 Orstad SL, McDonough MH, Stapleton S, et al. A systematic review of agreement between perceived and objective neighborhood environment measures and associations with physical activity outcomes. Environ Behav 2017;49:904‒932.

11 Kim DH, Yoo S. How does the built environment in compact metropolitan cities affect health? a systematic review of Korean studies. Int J Environ Res Public Health 2019;16(16):2921. doi: 10.3390/ijerph16162921

12 Ma L, Dill J. Associations between the objective and perceived built environment and bicycling for transportation. J Transp Health 2015;2(2):248‒255.

13 Yamaguchi M, Praditsorn P, Purnamasari SD, et al. Measures of perceived neighborhood food environments and dietary habits: A systematic review of methods and associations. Nutrients 2022;14(9):1788.

For peer review only - http://bmjopen.bmj.com/site/about/guidelines.xhtml

14 Kang CD. The S+ 5Ds: spatial access to pedestrian environments and walking in Seoul, Korea. Cities 2018;77:130–141.

15 Sawitri DR, Hadiyanto H, Hadi SP. Pro-environmental behavior from a socialcognitive theory perspective. Procedia Environ Sci 2015;23:27–33.

16 Cope M, Elwood S. Qualitative GIS: A Mixed Methods Approach. London: SAGE Publications 2009:1–12.

17 Mennis J, Mason MJ, Cao Y. Qualitative GIS and the visualization of narrative activity space data. Int J Geogr Inf Sci 2013;27(2):267–291.

18 Kim J, Kim DH, Lee J, et al. A scoping review of qualitative geographic information systems in studies addressing health issues. Soc Sci Med 2022;314:115472.

19 Yoo S, Kim DH. Perceived urban neighborhood environment for physical activity of older adults in Seoul, Korea: a multimethod qualitative study. Prev Med 2017;103:S90–S98.

20 Gwanak-gu office. General status of Gwanak-gu [online]. 2022. https://www.gwanak.go.kr/site/gwanak/11/11101010000002016051207.jsp (accessed 15 December 2022).

21 Park SY. Housing poverty in Korea. Health and Welfare Policy Forum 2012;2012(2):33-46. doi: 10.23062/2012.02.5.

22 Guest G, Bunce A, Johnson L. How many interviews are enough? an experiment with data saturation and variability. Field Methods 2006;18(1): 59–82.

23 Hennink M, Kaiser BN. Sample sizes for saturation in qualitative research: a systematic review of empirical tests. Soc Sci Med 2022;292:114523.

24 Legard R, Keegan J, Ward K. In-depth interviews. In: Qualitative Research Practice: A Guide for Social Science Students and Researchers. London: SAGE Publications 2003:138–169.

25 Mulhall A. In the field: notes on observation in qualitative research. J Adv Nurs 2003;41(3):306–313.

26 Lambton Public Health. Built Environment Healthy Community Checklist [online]. 2017. https://lambtonpublichealth.ca/resource/built-environment-healthy-community-checklist-2017/ (accessed 31 October 2022).

27 Moeser SD. Cognitive mapping in a complex building. Environ Behav 1988;20(1):21–49.

28 Jeong, GS, Kong SH. GIS based advanced positioning technique for mobile GPS. JKICS 2015;40(11):2261–2270.

For peer review only - http://bmjopen.bmj.com/site/about/guidelines.xhtml

29 Zenk SN, Matthews SA, Kraft AN, Jones KK. How many days of global positioning system (GPS) monitoring do you need to measure activity space environments in health research? Health Place 2018;51:52–60.

30 Braun V, Clarke V. Using thematic analysis in psychology. Qual Res Psychol 2006;3(2):77–101.

31 Downe-Wamboldt, B. Content analysis: method, applications, and issues. Health Care Women Int 1992;13(3):313–321.

32 Jung JK, Elwood S. Extending the qualitative capabilities of GIS: computer-aided qualitative GIS. Trans GIS 2010;14(1):63–87.

33 Smit B. Introduction to ATLAS.ti for mixed analysis. In: The Routledge Reviewer's Guide to Mixed Methods Analysis. New York: Routledge 2021:331–342.

For peer review only

For peer review only - http://bmjopen.bmj.com/site/about/guidelines.xhtml

FIGURE CAPTIONS

FIGURE 1 Schematic diagram of this study

FIGURE 2 Study site

FIGURE 3 Data collection process

For peer review only

For peer review only - http://bmjopen.bmj.com/site/about/guidelines.xhtml

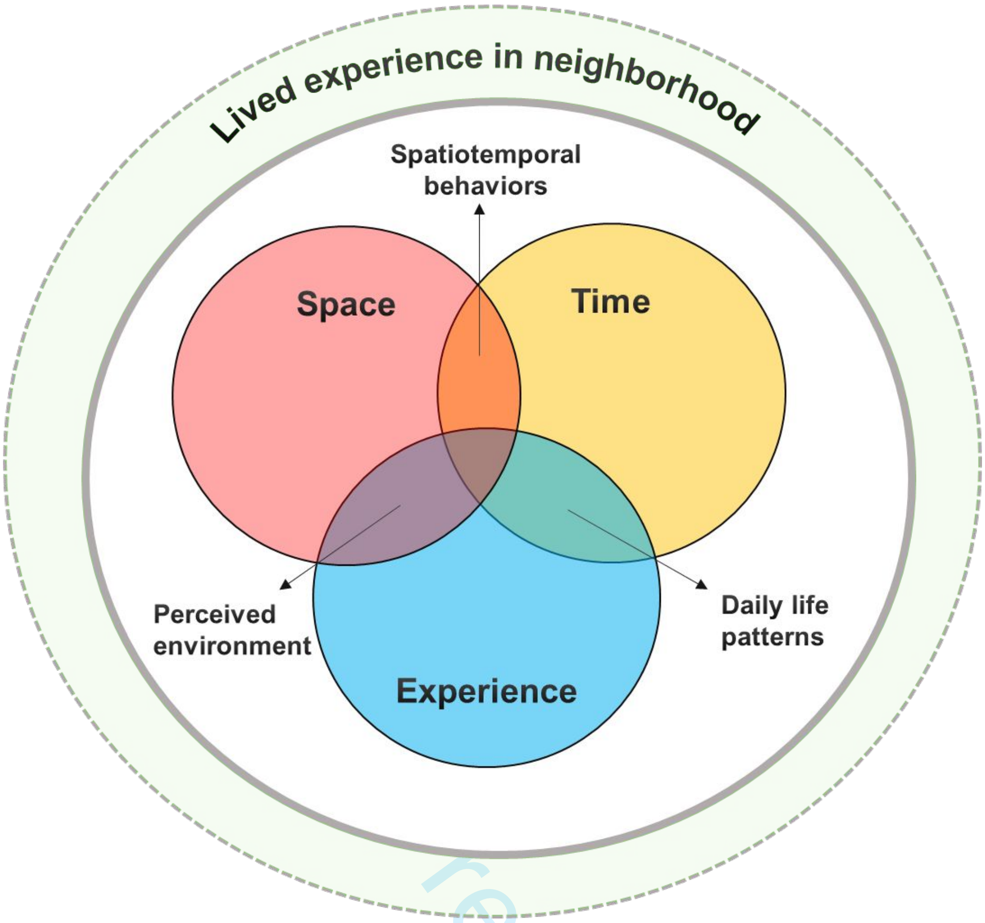

FIGURE 1 Schematic diagram of this study

For peer review only

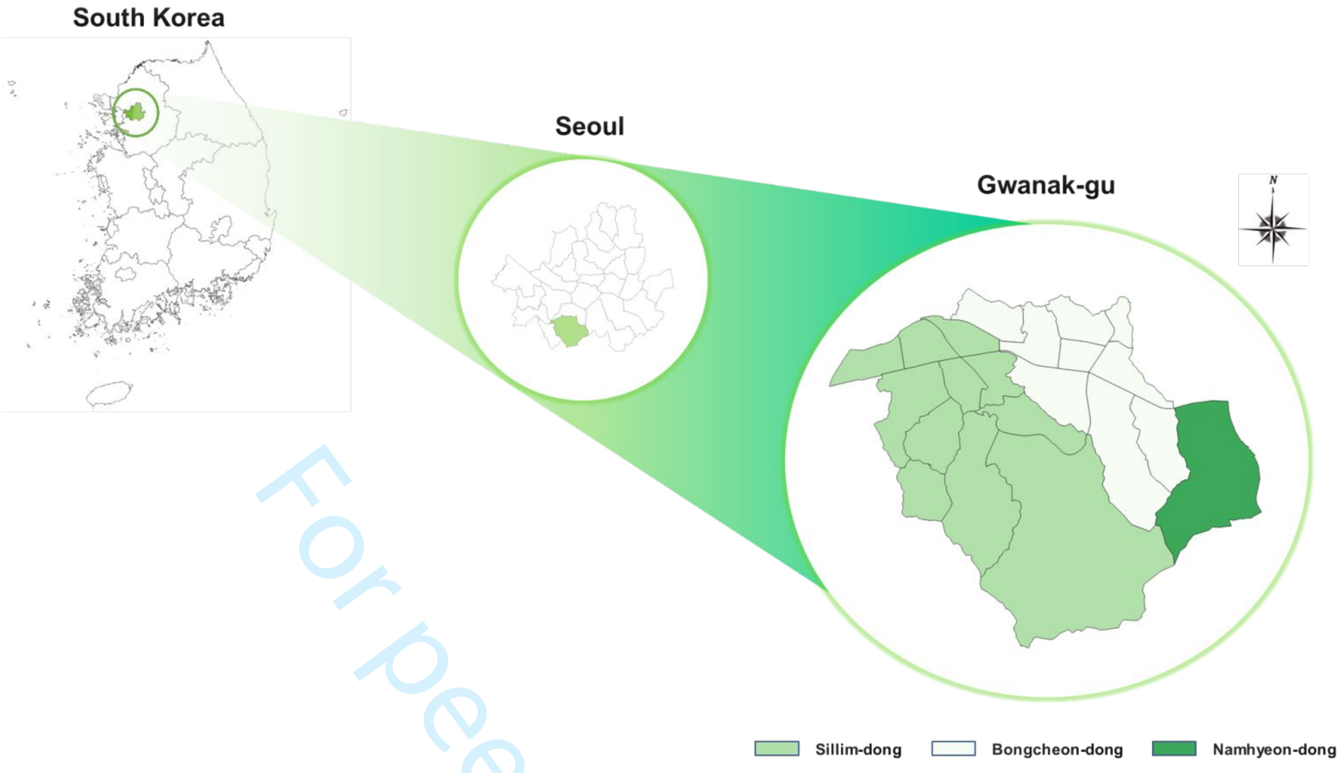

FIGURE 2 Study site

For peer review only

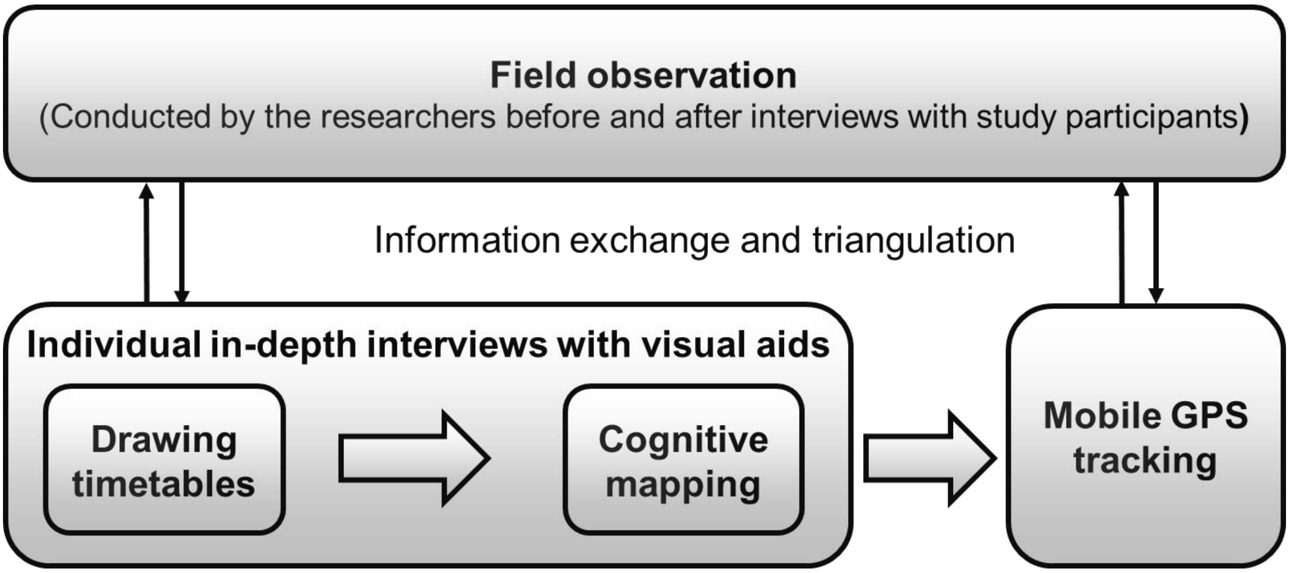

FIGURE 3 Data collection process

For peer review only

SUPPLEMENT MATERIAL 1. Interview questions

Activities	Interview questions
Prepare a timetable (Confirmation by displaying on the timetable based on participant responses)	**1. We'd like to draw a timeline of your daily life. Describe a typical day in your life.**<br>- How long has this lifestyle been going on?<br>- Is there a reason for this change in lifestyle (if it's different before or after COVID-19)?<br><br>**2. We'd like to ask your thoughts on "healthy living".**<br>- What do you think "healthy living" is about practicing or enjoying in your daily life?<br>- As you reflect on your day, describe any activities you maintain or try to do to stay healthy.<br>- If there's something you'd like to try to do for your health and you're not able to, please explain what you can't do and why.<br>- Thinking about your day, describe any activities that you do that contribute to poor health.<br>- Describe any reasons why you engage in the activity despite its health risks.
Prepare a neighborhood map (Confirm by displaying on a map based on participant responses)	**3. We would like to ask you about places, spaces that you associate with 'healthy living'.**<br>- Looking at the map, please explain where you do/would like to do each of the activities related to "healthy living" that you mentioned earlier.<br>- Where is this space/place located? Please mark it on the map.<br>- What activities do you typically do in that space/location?<br>- Why did you choose that space/location, if any?<br>- What are the challenges of utilizing that space/location?<br>(*Need to check: the environment of your current home)
	**4. We'd like to ask you about your neighborhood's health-related environment.**<br>- Fill in the blanks in the sentence "One thing about my neighborhood, [ ], affects my health" and be specific about why.<br>- Show me on a map where you think my neighborhood exemplifies the characteristics you just described.<br>- Plot the route from your home to the location on a map.<br>- If you've been to or used these places, please describe what you

For peer review only

	did there, for what purpose, and what it was like.<br>- If there's one thing you'd like to see improved in your neighborhood for healthy living, please describe it.<br>- Assuming you live in the neighborhood you live in now for a long time to come, how do you think the meaning of your neighborhood or your use of it will change?<br>- Based on what we've talked about so far, plot on a map where you think your "neighborhood" is and where it ends.

For peer review only

SUPPLEMENT MATERIAL 2. Checklist of field observation (Lambton Public Health, 2017)

Check areas	Check points
**HEALTHY Transportation Networks**	HAVE YOU...<br>☐ Supported a variety of transportation modes that are convenient and safe for all ages?<br>☐ Encouraged retail and commercial spaces that support active transportation with connections to cycling paths, trails, sidewalks or transit, if applicable?<br>☐ Required or encouraged homes and places of employment to be within a comfortable walking or biking distance of frequent destinations such as schools, parks, shops, and transit stops, if applicable?<br>☐ Designed roadways and access to buildings and services as pedestrian friendly
**HEALTHY Food Systems**	HAVE YOU...<br>☐ Supported and provided space to grow food (agriculture or neighborhood gardens)?<br>☐ Supported access to healthy and local food?<br>☐ Supported the integration of all of the pieces of the food system in your municipality to enhance the environmental, economic, social, and nutritional health of the community (Producing, Processing, Distributing, Accessing Food, Eating, and Managing Waste)?
**HEALTHY Neighborhood Design**	HAVE YOU...<br>☐ Encouraged mixed land-use designations in your community?<br>☐ Adequately distributed trails, parks or recreation facilities in your community?<br>☐ Promoted or designed your community where people can easily connect with each other and a variety of day-to-day services?<br>☐ Encouraged the Three Cs of healthy neighborhoods: Complete, Compact and Connected?<br>☐ Examined hazard, safety and vandalism concerns in your community
**HEALTHY Natural Environments**	HAVE YOU...<br>☐ Made parks and natural areas of your community accessible to all demographics, safe and connected?

For peer review only

	☐ Protected and enhanced natural areas from development?<br>☐ Incorporated trees and natural features into all new and re-developments, including road work?<br>☐ Incorporated trees and natural features to reduce UV exposure, air pollution, water pollution and urban heat islands?
**HEALTHY Housing**	HAVE YOU...<br>☐ Promoted affordable housing programs?<br>☐ Regulated and enforced housing quality standards?<br>☐ Addressed any high frequencies of tenant/resident relocation or turnover?<br>☐ Designated housing within close proximity to other services and away from environmental hazards?

For peer review only

SUPPLEMENT MATERIAL 3. Round-shaped timetables

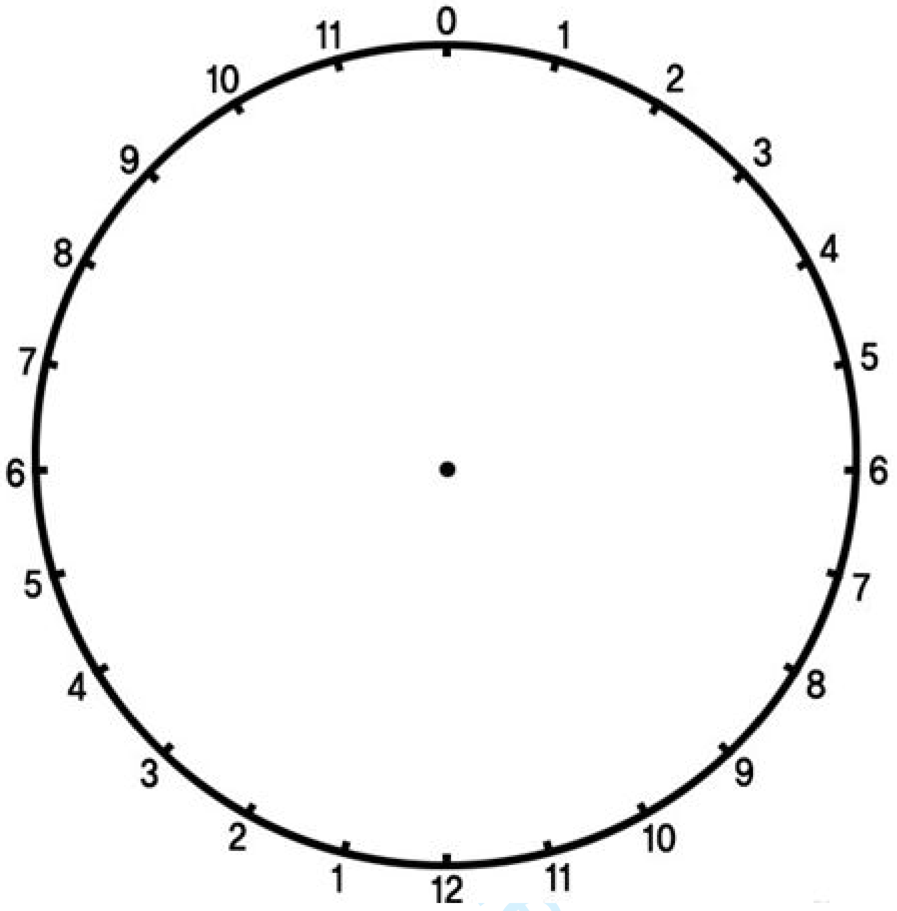

For peer review only - http://bmjopen.bmj.com/site/about/guidelines.xhtml

BMJ Open

Neighborhood environments for a healthy lifestyle among young single-person households experiencing housing poverty in Seoul, South Korea: A spatiotemporal qualitative study protocol

Journal:	*BMJ Open*
Manuscript ID	bmjopen-2023-077234.R1
Article Type:	Protocol
Date Submitted by the Author:	20-Nov-2023
Complete List of Authors:	Kim, Dong Ha; Seoul National University, Institute of Health and Environment<br>Lee, Jihyun; Seoul National University, Department of Public Health Sciences, Graduate School of Public Health<br>Yoo, Seunghyun; Seoul National University, Department of Public Health Sciences, Graduate School of Public Health; Seoul National University, Institute of Health and Environment
**Primary Subject Heading**:	Qualitative research
Secondary Subject Heading:	Public health, Research methods
Keywords:	Health Equity, PUBLIC HEALTH, QUALITATIVE RESEARCH, Quality of Life

SCHOLARONE™
Manuscripts

Neighborhood environments for a healthy lifestyle among young single-person households experiencing housing poverty in Seoul, South Korea: A spatiotemporal qualitative study protocol

ABSTRACT

Introduction

The number of single-person households is increasing globally—including in South Korea, where they account for over 30% of all households. Young single-person households in South Korea face health problems and housing challenges. Both the perceived and objective aspects of the neighborhood environment, as a community asset, play a significant role in sustaining a healthy lifestyle. This study aims to explore and describe the meaning, roles, and spatiotemporal characteristics of neighborhood environments for a healthy lifestyle in young single-person households experiencing housing poverty in Seoul, South Korea.

Methods and analysis

This ongoing study uses an extended qualitative geographic information systems (GIS) approach to explore a district in the city of Seoul that has the highest population density of young single-person households experiencing housing poverty. The study sample comprises young single-person households aged 19–39 years who are experiencing housing poverty in the study area, with an expected saturation point of approximately 55 participants. We employ online and offline recruitment strategies to ensure the inclusion of diverse perspectives and a multimethod approach that combines descriptive and spatiotemporal data collection techniques (e.g., individual in-depth interviews, field observations, and mobile global positioning system (GPS) tracking). The data analysis encompasses thematic and content analyses to understand the neighborhood environment's perceived attributes and the spatiotemporal characteristics of healthy lifestyles. In the integrated analysis, we plan to combine the qualitative findings with living space and daily-life patterns using qualitative software and a hybrid relational database.

Ethics and dissemination

The Institutional Review Board approved the research protocol on May 18, 2021. The findings will be shared at international conferences and published in academic journals. Additionally, an online seminar will be conducted to share the results with policymakers, researchers, community organizations, and health workers working with

For peer review only - http://bmjopen.bmj.com/site/about/guidelines.xhtml

For peer review only

young single-person households experiencing housing poverty.

Strengths and limitations of this study

- This study provides valuable insights by exploring the lived experiences of young single-person households experiencing housing poverty.
- This study helps identify the meanings and values attached to young single-person households experiencing housing poverty in various places and to understand how these meanings are formed and interpreted.
- This study may be time-consuming, labor-intensive, and costly due to the need to collect and analyze data from multiple sources and devices.
- This study requires a high level of competence and expertise in managing data quality to address the complexities of integrating diverse data sources and ensure the active participation of the target population.
- The interpretation of the study results should consider the characteristics and context of both the study area and participants, particularly in relation to housing poverty.

INTRODUCTION

Single-person households are becoming increasingly common worldwide. In European Union member states, the proportion of single-person households increased from 31% in 2010 to 34% in 2017 (1). According to the United States Census Bureau (2), the proportion of single-person households in the United States increased from 16.7% in 1969 to 28.4% in 2019, and the rate of increase is expected to accelerate over the next decade. Similar to most developed countries, South Korea (hereafter, Korea) has experienced notable household fragmentation in recent years, leading to a trend toward individualized life courses. In 2000, single-person households accounted for only 15.5% of all households in Korea, but had the highest household composition ratio (27.2%) and overtook multi-person households in 2015. This figure had risen even further to 33.4% by 2021 (3).

Compared to the other demographic groups in Korea, young single-person households face a higher risk of health and housing in 2020, 63.2% of young single-person households aged 19–29 were either obese or overweight, 27.7% showed symptoms of depression, and 29.3% were smokers (4). These figures were statistically significantly

For peer review only

For peer review only - http://bmjopen.bmj.com/site/about/guidelines.xhtml

higher than those for multi-person households (5). In 2021, the Korea Research Institute for Human Settlements (6) reported that 60% of young single-person households spent more than 30% of their monthly income on housing rent, which is than five times higher than that of young couple households and households with young adults living with parents. Additionally, according to the 2020 Korea Housing Survey (7), 9.6% of single-person households living in houses with less than 14 m^2 of residential space experienced difficulties in soundproofing, ventilation, heating, and kitchen facilities. These housing conditions are lacking compared to the international minimum housing standards that provide specific facilities and layouts such as kitchens, bathrooms, privacy protections, appropriate hot water supply temperatures, and 25 m^2 of space per person (8). This suggests that young single-person households in Korea may not have sufficient resources within their homes to manage and protect their health adequately. Given the challenges faced by these households, namely, insufficient home resources to adequately address health needs, highlighting the health-promoting factors of the neighborhood environment could be a pivotal strategy for improving the quality of life of those living in single-person households.

Neighborhood environment refers to the physical, social, economic, and cultural factors that surround and influence residents within a specific geographic area (9). These factors include land use patterns, street safety, public transportation systems, living facilities, parks and green spaces, housing quality, and social and cultural opportunities. Additionally, the neighborhood environment has perceived and objective aspects (10). The *perceived aspect* of a neighborhood environment refers to how individuals perceive and experience their neighborhood (11). In contrast, the *objective aspect* of the neighborhood environment refers to the physical and structural characteristics of the neighborhood that can be measured quantitatively (11). The perceived and objective aspects of the neighborhood environment are significant in shaping individuals' experiences and health outcomes. Perceived aspects influence how individuals behave and engage with their communities, whereas objective aspects affect access to resources and services, safety, and overall well-being (12). By understanding the perceived and objective aspects of the neighborhood environment, policymakers, researchers, and community members can collaborate to create healthier and more livable neighborhoods.

Collecting data on various aspects, such as time, space, and experience, is crucial for comprehensively understanding the perceived and objective neighborhood environments related to healthy lifestyles. The time available for utilizing the neighborhood environment can shape patterns can form a pattern of participation in

For peer review only

health behaviors (13), and spatial access to neighborhood environments can affect an individual's ability to maintain a healthy lifestyle (14). Perceptions of neighborhood environments can also affect intentions, practices, and attitudes toward health behaviors (15). Therefore, considering these aspects in data collection can provide a more comprehensive understanding of the relationship between the neighborhood environment and healthy lifestyle of young single-person households experiencing housing poverty.

This study aims to explore and describe the meaning, roles, and spatiotemporal characteristics of neighborhood environments in order to secure healthy lifestyles for young single-person households experiencing housing poverty in Seoul, Korea. The specific research questions are as follows:

1) What perspectives do young single-person households experiencing housing poverty have on their homes and neighborhoods in terms of what would promote a healthy lifestyle?

2) How do young single-person households experiencing housing poverty maintain or attempt to pursue a healthy lifestyle in their neighborhoods?

3) What positive attributes and characteristics of neighborhood environments are necessary to support healthy lifestyles in young single-person households experiencing housing poverty?

4) What is the context and scope of perceived neighborhood boundaries for young single-person households experiencing housing poverty?

METHODS AND ANALYSIS

Study design

This study employs an extended qualitative GIS (QGIS) research design by adding time-use data from daily life. The original QGIS methodology integrates qualitative and geospatial methods to investigate the rich contextual details of spatial experiences in various formats (16). By merging qualitative methods with GIS-based techniques, QGIS provides an analytical framework for narrative inquiries into spatial experiences that have previously been unfeasible (17, 18). One advantage of visualizing textual information on a map (e.g., perceptions, attitudes, emotions, beliefs, and behaviors) is the ability to explore spatial and environmental contexts that may not be fully elucidated through interviews alone (19). By incorporating time aspects into these benefits, we expect to explore the daily time usage patterns of young single-person households experiencing housing poverty in their

For peer review only - http://bmjopen.bmj.com/site/about/guidelines.xhtml

neighborhoods.

Figure 1 shows a schematic diagram of the extended QGIS used in this study. This diagram demonstrates how qualitative, spatial, and temporal data can be combined through simultaneous processing to produce a rich contextual understanding of people's lived experiences in their neighborhoods. This distinctive aspect sets our study's strategy apart from a mixed methods approach (20) as it has a unique sequence and arrangement for collecting both qualitative and quantitative data. Additionally, we take a more qualitative-oriented approach compared to traditional QGIS by interpreting GIS information based on patterns rather than concentrating solely on size, frequency, and density.

[Figure 1]

Setting

Our study is currently being conducted in an administrative district with the highest density of young single-person households experiencing housing poverty, Gwanak-gu, which is located in southern Seoul (Figure 2). The district has three administrative neighborhoods with a total area of 29.6 km^2, 60% of which is occupied by mountainous terrain (21). As of September 2021, there were 164,000 single-person households in the district, making it the region with the largest number of single-person households in Korea (21). The main housing type in the district is the multiple complex house with a semi-basement, accounting for 55% of the total houses (21). The average per capita housing area was 28.2 m^2 (approximately 303 ft^2), and the monthly rent for a studio apartment was about 400,000 won (approximately 299.00 USD) in 2021. Gwanak-gu is known for its poor and low-cost residential environment (21). In addition, the district has a low-income community ("Go-si-chon" in Korean) where a large number of unemployed young people who prepare for the civil service examinations live in close quarters (21).

[Figure 2]

Study sample and recruitment

Three administrative neighborhoods in Gwanak-gu, where young single-person households are concentrated, are included in our research as study areas. We are currently in the planning phase of conducting stratified purposive sampling (22) for single-person households (aged 19–39) experiencing housing poverty within the

For peer review only - http://bmjopen.bmj.com/site/about/guidelines.xhtml

study areas. In this study, housing poverty is defined as residing in housing that either 1) fails to meet the minimum housing standards in Korea, or 2) requires more than 30% of and individual's monthly household income (23). The exclusion criteria are individuals who have lived in the study areas for less than six months and those who do not possess electronic and digital devices capable of conducting online interviews. Online and offline recruitment strategies are currently being employed to ensure the inclusion of diverse perspectives from participants of various genders, ages, employment statuses, housing types, and residence periods. Online recruitment involves posting recruitment documents on social network service (SNS) platforms commonly used by young people in the study areas with the cooperation of online community managers. Offline recruitment entails attaching recruitment documents to telephone poles, bulletin boards, and walls located in areas frequented by local young people, as well as visiting public service centers that rent spaces to local young people. Participants will be recruited until data saturation is achieved; that is, when new information no longer produces significant changes to the code book (24). The saturation point of study participants is expected to be approximately 9–17 people per neighborhood, based on previous research (25), and with additional participants to confirm saturation, the number of study participants is expected to be approximately 55.

Data collection

We are currently employing a multi-method approach to gather descriptive and spatiotemporal information on the neighborhood environment to determine what would be conducive for healthy lifestyles among young single-person households experiencing housing poverty. This approach combines descriptive data collection techniques, such as individual in-depth interviews, spatial data collection techniques (e.g., cognitive mapping, field observations, and mobile GPS tracking), and temporal data collection techniques (e.g., drawing round-shaped timetables, which are hourly schedules represented in a circular format). The data collection process involves both non-human research (field observations) and human subject research. We are conducting human subject research in the order of individual in-depth interviews using cognitive maps and round-shaped timetables, followed by mobile GPS tracking (Figure 3). Data collection has been ongoing since September 2022.

[Figure 3]

Descriptive data collection

Individual in-depth interviews are an effective and proactive qualitative research method that can be used to

For peer review only - http://bmjopen.bmj.com/site/about/guidelines.xhtml

understand the lives and experiences of research participants in their language and perspective and to discover their inner world (26). Different types of individual in-depth interviews can be conducted depending on the means, number of research participants, interview format, and openness to interview questions. The most well-known types include open, semi-structured, and structured interviews. For this ongoing study, we are conducting semi-structured interviews with young single-person households experiencing housing poverty. The interviews are being conducted face-to-face and through the online videoconferencing platform Zoom, depending on participants' preferences. The researchers first explain the study and then obtain consent from individuals regarding their willingness to participate. Additionally, they confirm their consent to record and use their interviews, which will be transcribed and used only for the purpose of analysis. Participants are informed that they can stop the interview at any time if they wish, and the informed consent form makes it clear that participation is entirely voluntary.

After obtaining consent, we conduct a survey to collect demographic information, including sex, age, occupation, length of residence, housing characteristics, and health status. Following the survey, we conduct one-on-one in-depth interviews using semi-structured questions to gain insights into the perceived attributes of neighborhood environments related to healthy lifestyles (Supplement Material 1). We record and transcribe the interviews into electronic documents with an average turnaround time of five days. The expected average interview time per participant is approximately one hour and 30 minutes.

Spatial data collection

Field observation is a spatial research method in which a researcher observes phenomena related to a research topic while moving around a research site (27). Currently, we are following a procedure for conducting field observations. We use Google Maps and Street View to examine the neighborhood environment and plan observation paths, schedules, and boundaries. To investigate the physical characteristics of the neighborhood environment and the contents that change depending on the observation time, we use The Healthy Community Checklist (28). We are manually recording the collected information on field observation sheets (Supplement Material 2), which will later be digitized for future reference and organized by date and neighborhood.

Techniques to visually represent an individual or group's mental model of the physical environment are known as cognitive mapping (29), which involves asking participants to either map from their memories or annotate existing maps with information that reflects their perceptions, knowledge, and experiences of the

For peer review only - http://bmjopen.bmj.com/site/about/guidelines.xhtml

environment. In this study, the researchers conduct activities during one-on-one interviews with the participants to create cognitive maps. Participants are asked to mark the map with reference to places and experiences related to healthy living. Upon completion of the cognitive map, the researchers ask the participants to explain the main activities in the marked places, their reason for selecting them, and any difficulties they encountered while using them. Finally, participants are asked to indicate the environmental characteristics of their neighborhood (within what they perceive to be its boundaries) that impact their health. We expect cognitive maps to provide insights into how people navigate and use their environments, perceive and prioritize different features and landmarks, and conceptualize their spatial relationships.

Mobile GPS can easily track the spatial movement of participants' daily activity radius in real-time (30). In this study, the participants wear the Garmin Forerunner device and record their daily lives during the survey period. The mobile GPS devices help confirm the range of participants' walking activity radii and identify their movement paths and the locations where they stayed in the neighborhood. The participants' data, including distance traveled, duration of movement, movement path, and place of residence are automatically synchronized with Google Maps and stored daily. Based on previous research evidence the research team conducts the tracking once for a period of three days, including two weekdays and one day on the weekend (31).

Temporal data collection

During the one-on-one in-depth interviews, we use a technique that creates a round-shaped timetable to understand participants' 24-hour daily routines, which are separated into weekdays and weekends. We divide a 24-hour day into equal segments and assign activities or events to each segment based on the start and end times. Each segment is labeled and represented by a circle, similar to a clock face (Supplemental Material 3). We ask participants to complete a daily schedule based on weekday and weekend routines. Once completed, the researchers review participants' schedules and ask for additional explanations of their daily neighborhood activities. Finally, we ask participants to describe activities related to a healthy lifestyle and explain why, when, and how often they engaged in them. We expect the round timetable to outline how individuals allocate their time, the duration and frequency of different activities, and the temporal patterns of their behavior.

Data analysis and integration

We will conduct six stages of thematic analysis (32) for the in-depth interview data. In the first stage, we

For peer review only - http://bmjopen.bmj.com/site/about/guidelines.xhtml

will develop a general framework for participants' perceptions of the neighborhood environment related to a healthy lifestyle by repeatedly reading the interview data. In the second stage, we will conduct an open coding process to identify all meaning units related to the perception of the neighborhood environment, which affects participants' healthy lifestyles, such as meaning, attributes, and roles. In the third stage, we will compare and contrast the relationships between the generated codes to identify categories. In the fourth stage, we will review the tentatively derived themes to determine whether they match the research purpose and reflect the data content. In the fifth stage, we will refine the derived themes into precise definitions through a recursive process of returning data for comparative analysis. Based on the final analysis results, we will provide a detailed description and present appropriate explanations and discussions on the research purpose.

We plan to perform content analysis using spatiotemporal data. Content analysis offers a systematic and objective approach to draw accurate inferences from verbal, visual, or written data, thus enabling the description and quantification of specific phenomena (33). We will extract information, including time usage patterns (amount of time and time of day), activity types, and activity range (within/outside the neighborhood), from the timetables created by individual study participants and analyze this information through overlapping analysis. We will convert the cognitive map data provided by participants and the field observation data surveyed by the researchers into a digital map using the Quantum GIS program in order to quantitatively analyze spatial characteristics (location, density, radius, distance, and distribution). We will analyze the mobile log data collected through mobile GPS tracking for neighborhood mobility patterns using the statistical software R and Google Maps.

During the integrated analysis phase, we plan to combine the qualitative results with living space and daily life pattern results using Atlas.ti qualitative software and a hybrid relational database. The hybrid relational database will allow us to create a descriptive data database under the attribute table of spatiotemporal data, which can integrate various types of qualitative data, such as images, texts, and videos, into spatial data (34). Atlas.ti can process text data and non-text multimedia data and be linked to GIS and codes (35).

Researcher characteristics and reflexivity

The research team consists of individuals aged 30 and above who are majoring in public health sciences at the university in the study area. This composition enhances the team's understanding of the neighborhoods and facilitates participant recruitment. However, it is worth noting that none of the researchers belong to the

demographic of young adults living in housing poverty, thus potentially introducing bias or leading to an overestimation in reading and interpreting the context of these young adults' lives.

Trustworthiness

We will apply Lincoln and Guba's (1985) criteria (36) for rigor in qualitative research (credibility, transferability, dependability, and confirmability) to enhance the trustworthiness of this study. To enhance the credibility of our research, we plan to cross-verify the perspectives, experiences, and behaviors of individuals collected from various sources using multiple qualitative methods. To improve transferability, we will provide a detailed description of the phenomenon, enabling a comparison with background data to establish the study's context. We will offer an in-depth methodological description of the study, which promotes replicability and enhances dependability. Additionally, the study will be audited by external experts with many years of experience in qualitative research to enhance confirmability.

Reporting of findings

The findings of this qualitative study will be reported in accordance with the Standards for Reporting Qualitative Research (SRQR) statement (37). The application of SRQR is not confined to the writing phase alone; these standards can aid researchers in planning qualitative studies and in meticulously documenting processes and decisions made throughout the study (Supplementary Material 4).

Patient and public involvement

There is no patient and public involvement in the design, conduct, reporting, or dissemination plans of this study. However, we obtain advice from key informants in the study area, including public service providers and long-term residents, to ensure pathways for participant recruitment and to enhance the research team's understanding of the local context.

ETHICS AND DISSEMINATION

The Institutional Review Board of the researchers' affiliated institution approved the research on May 18, 2021 (IRB No. 2106/002-018). Before participating in the study, participants are asked to provide written informed consent to ensure complete anonymity and confidentiality. The informed consent form explains in detail the purpose of the recording, possible risks and discomfort, potential benefits, confidentiality of information, and

For peer review only - http://bmjopen.bmj.com/site/about/guidelines.xhtml

withdrawal procedures. Participants' anonymity is protected, and no identifiable characteristics are specified in the recorded conversations. We will disseminate the findings to the academic society by presenting them at international conferences on urban health and publishing them in academic journals. In addition, we plan to conduct an online seminar/webinar to share and discuss our research findings with policymakers, researchers, community organizations, and health workers who plan and implement policies and programs for young single-person households experiencing housing poverty.

Contributors D.H.K., J.L., and S.Y. conceived the study. D.H.K., J.L., and S.Y. contributed to the development of the study design and final protocols for sample selection and data collection. D.H.K. and J.L. contributed to writing the manuscript. S.Y. provided critical editing and approved the final version of the paper.

Funding This work was supported by a National Research Foundation (NRF) grant funded by the Korean government (Ministry of Science, ICT, and Future Planning; NRF- 2020R1A2C2012463).

Competing interests None declared.

Patient and public involvement Patients and/or the public were not involved in the design, or conduct, or reporting, or dissemination plans of this protocol.

Patient consent for publication Not required.

Provenance and peer review Not commissioned; externally peer reviewed.

For peer review only - http://bmjopen.bmj.com/site/about/guidelines.xhtml

REFERENCES

1 Eurostat. Eurostat Regional Yearbook: 2019 Edition. Eurostat Publications Office 2019. doi: 10.2785/1522

2 United States Census Bureau. One-person households on the rise [online]. 2019. https://www.census.gov/library/visualizations/2019/comm/one-person-households.html (accessed 7 April 2023).

3 Statistics Korea. Single-Person Households in 2021 Statistics (Korean). Statistics Korea 2021.

4 Ministry of Health and Welfare, Korea Disease Control and Prevention Agency. Announcement of the Results of the 8th National Health and Nutrition Survey for the 2nd Year. 2020.

5 Shin, MA. Comparative study on health behavior and mental health between one person and multi-person households: Analysis of data from the National Health and Nutrition Examination Surveys (Korean). KSW 2019;14(4):11-23.

6 Park M, Woo J. Complementary strategies for housing vulnerability by age group of single-person households. Sejong: Korea Research Institute for Human Settlements 2021:16–18.

7 Korea Research Institute for Human Settlements. 2020 Housing Survey. Sejong: Ministry of Land, Infrastructure and Transport 2020:91–92.

8 Yoo D, Lee J, Jang E. An international comparative study to shift from minimum to decent housing standard (Korean). JAIK 2022;38:47–58.

9 Ding D, Sallis JF, Kerr J, et al. Neighborhood environment and physical activity among youth: A review. Am J Prev Med 2011;41:442–55. doi: 10.1016/j.amepre.2011.06.036.

10 Orstad SL, McDonough MH, Stapleton S, et al. A systematic review of agreement between perceived and objective neighborhood environment measures and associations with physical activity outcomes. Environ Behav 2017;49:904–932.

11 Kim DH, Yoo S. How does the built environment in compact metropolitan cities affect health? A systematic review of Korean studies. Int J Environ Res Public Health 2019;16(16):2921. doi: 10.3390/ijerph16162921

12 Ma L, Dill J. Associations between the objective and perceived built environment and bicycling for transportation. J Transp Health 2015;2(2):248–255.

For peer review only - http://bmjopen.bmj.com/site/about/guidelines.xhtml

13 Yamaguchi M, Praditsorn P, Purnamasari SD, et al. Measures of perceived neighborhood food environments and dietary habits: A systematic review of methods and associations. Nutrients 2022;14(9):1788.

14 Kang CD. The S+ 5Ds: spatial access to pedestrian environments and walking in Seoul, Korea. Cities 2018;77:130–141.

15 Sawitri DR, Hadiyanto H, Hadi SP. Pro-environmental behavior from a social-cognitive theory perspective. Procedia Environ Sci 2015;23:27–33.

16 Cope M, Elwood S. Qualitative GIS: A mixed methods approach. London: SAGE Publications 2009:1–12.

17 Mennis J, Mason MJ, Cao Y. Qualitative GIS and the visualization of narrative activity space data. Int J Geogr Inf Sci 2013;27(2):267–291.

18 Kim J, Kim DH, Lee J, et al. A scoping review of qualitative geographic information systems in studies addressing health issues. Soc Sci Med 2022;314:115472.

19 Yoo S, Kim DH. Perceived urban neighborhood environment for physical activity of older adults in Seoul, Korea: A multimethod qualitative study. Prev Med 2017;103:S90–S98.

20 Creswell, JW. A concise introduction to mixed methods research. Thousand Oaks, CA: Sage Publications 2014:34-50.

21 Gwanak-gu office. General status of Gwanak-gu [online]. 2022. https://www.gwanak.go.kr/site/gwanak/11/11101010000002016051207.jsp (accessed 15 December 2022).

22 Patton MQ. Qualitative research and evaluation methods (3rd ed.). Thousand Oaks, CA: Sage 2002:240.

23 Park SY. Housing poverty in Korea (Korean). Health and Welfare Policy Forum 2012;2012(2):33-46. doi: 10.23062/2012.02.5.

24 Guest G, Bunce A, Johnson L. How many interviews are enough? An experiment with data saturation and variability. Field Methods 2006;18(1): 59–82.

25 Hennink M, Kaiser BN. Sample sizes for saturation in qualitative research: A systematic review of empirical tests. Soc Sci Med 2022;292:114523.

26 Legard R, Keegan J, Ward K. In-depth interviews. In: Qualitative Research Practice: A Guide for Social Science Students and Researchers. London: SAGE Publications 2003:138–169.

27 Mulhall A. In the field: Notes on observation in qualitative research. J Adv Nurs 2003;41(3):306–313.

28 Lambton Public Health. Built Environment Healthy Community Checklist [online]. 2017.

For peer review only - http://bmjopen.bmj.com/site/about/guidelines.xhtml

https://lambtonpublichealth.ca/resource/built-environment-healthy-community-checklist-2017/ (accessed 31 October 2022).

29 Moeser SD. Cognitive mapping in a complex building. Environ Behav 1988;20(1):21–49.

30 Jeong, GS, Kong SH. GIS-based advanced positioning technique for mobile GPS (Korean). JKICS 2015;40 (11):2261–2270.

31 Zenk SN, Matthews SA, Kraft AN, Jones KK. How many days of global positioning system (GPS) monitoring do you need to measure activity space environments in health research? Health Place 2018;51:52–60.

32 Braun V, Clarke V. Conceptual and design thinking for thematic analysis. Qual Psychol 2022;9(1): 3-6.

33 Downe-Wamboldt, B. Content analysis: method, applications, and issues. Health Care Women Int 1992;13 (3):313–321.

34 Jung JK, Elwood S. Extending the qualitative capabilities of GIS: Computer-aided qualitative GIS. Trans GIS 2010;14(1):63–87.

35 Smit B. Introduction to ATLAS.ti for mixed analysis. In: The Routledge Reviewer's Guide to Mixed Methods Analysis. New York: Routledge 2021:331–342.

36 Lincoln YS, Guba EG. Establishing trustworthiness. Naturalistic Inquiry 1985;289(331):289-327.

37 O'Brien BC, Harris IB, Beckman TJ, Reed DA, Cook DA. Standards for reporting qualitative research: A synthesis of recommendations. Acad Med 2014;89(9):1245-1251.

only

For peer review only - http://bmjopen.bmj.com/site/about/guidelines.xhtml

362 **FIGURE CAPTIONS**

363 **FIGURE 1 Schematic diagram of this study**

364 **FIGURE 2 Study site**

365 **FIGURE 3 Data collection process**

For peer review only

For peer review only - http://bmjopen.bmj.com/site/about/guidelines.xhtml

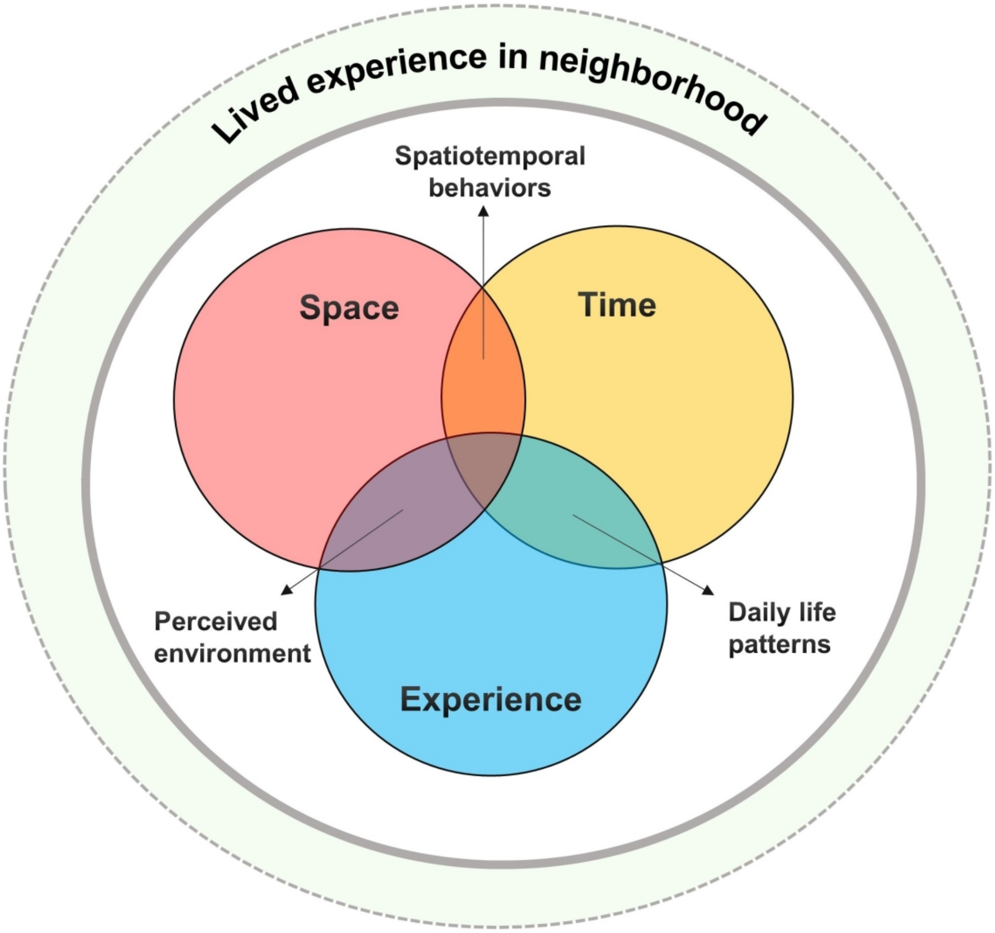

FIGURE 1. Schematic diagram of this study

185x173mm (300 x 300 DPI)

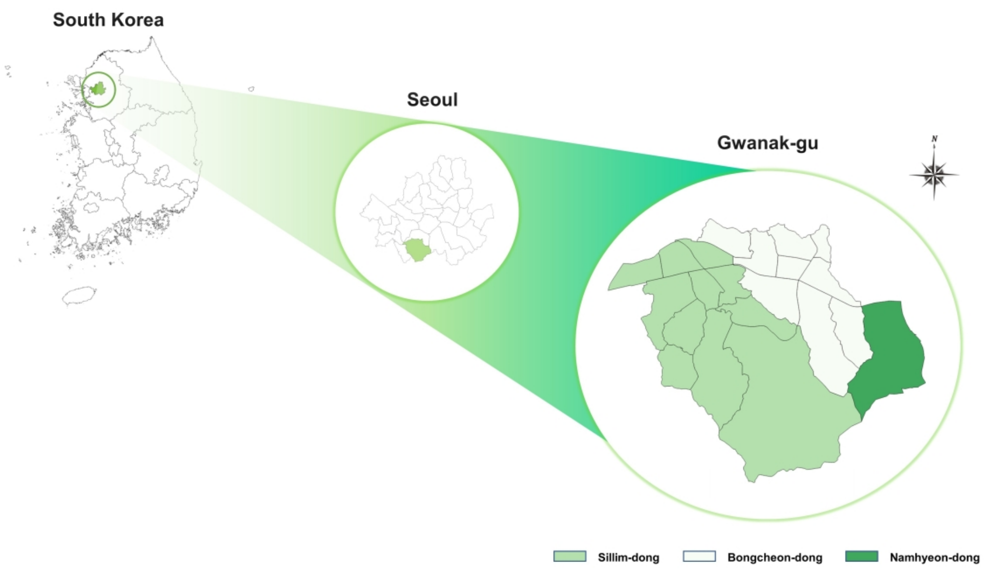

FIGURE 2. Study site

338x190mm (300 x 300 DPI)

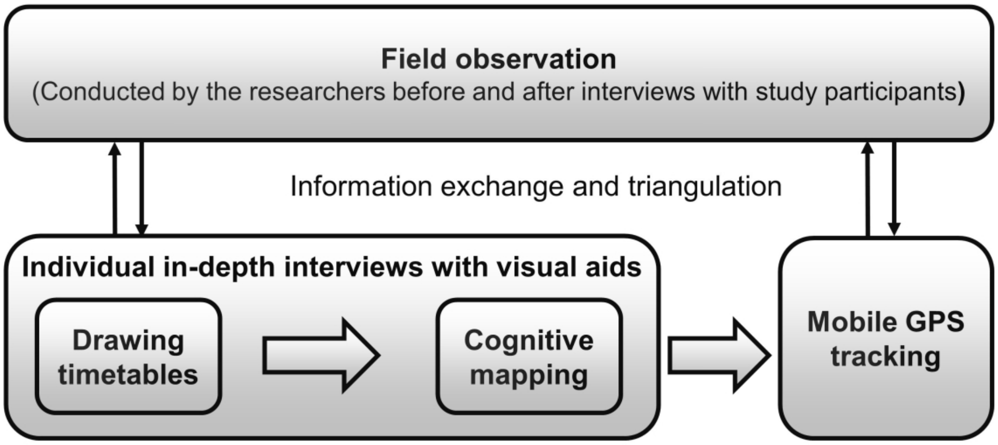

Who	What (Research activities)	When		
		Before the Interview	During the interview	After the interview
Researcher	Field observation			
Researcher & Participant	In-depth interview			
	Drawing timetables			
	Cognitive mapping			
Participant	Mobile GPS tracking			

FIGURE 3. Data collection process

246x182mm (300 x 300 DPI)

Supplement Material 1. Interview questions

Activities	Interview questions
Prepare a timetable (Confirmation by displaying on the timetable based on participant responses)	**1. We'd like to draw a timeline of your daily life. Describe a typical day in your life.**<br>- How long has this lifestyle been going on?<br>- Is there a reason for this change in lifestyle (if it's different before or after COVID-19)?<br><br>**2. We'd like to ask your thoughts on "healthy living".**<br>- What do you think "healthy living" is about practicing or enjoying in your daily life?<br>- As you reflect on your day, describe any activities you maintain or try to do to stay healthy.<br>- If there's something you'd like to try to do for your health and you're not able to, please explain what you can't do and why.<br>- Thinking about your day, describe any activities that you do that contribute to poor health.<br>- Describe any reasons why you engage in the activity despite its health risks.
Prepare a neighborhood map (Confirm by displaying on a map based on participant responses)	**3. We would like to ask you about places, spaces that you associate with 'healthy living'.**<br>- Looking at the map, please explain where you do/would like to do each of the activities related to "healthy living" that you mentioned earlier.<br>- Where is this space/place located? Please mark it on the map.<br>- What activities do you typically do in that space/location?<br>- Why did you choose that space/location, if any?<br>- What are the challenges of utilizing that space/location?<br>(*Need to check: the environment of your current home)
	**4. We'd like to ask you about your neighborhood's health-related environment.**<br>- Fill in the blanks in the sentence "One thing about my neighborhood, [ ], affects my health" and be specific about why.<br>- Show me on a map where you think my neighborhood exemplifies the characteristics you just described.<br>- Plot the route from your home to the location on a map.<br>- If you've been to or used these places, please describe what you

For peer review only

	did there, for what purpose, and what it was like.<br>- If there's one thing you'd like to see improved in your neighborhood for healthy living, please describe it.<br>- Assuming you live in the neighborhood you live in now for a long time to come, how do you think the meaning of your neighborhood or your use of it will change?<br>- Based on what we've talked about so far, plot on a map where you think your "neighborhood" is and where it ends.

For peer review only

Supplement Material 2. Checklist of field observation (Lambton Public Health, 2017)

Check areas	Check points
**HEALTHY Transportation Networks**	HAVE YOU...<br>☐ Supported a variety of transportation modes that are convenient and safe for all ages?<br>☐ Encouraged retail and commercial spaces that support active transportation with connections to cycling paths, trails, sidewalks or transit, if applicable?<br>☐ Required or encouraged homes and places of employment to be within a comfortable walking or biking distance of frequent destinations such as schools, parks, shops, and transit stops, if applicable?<br>☐ Designed roadways and access to buildings and services as pedestrian friendly
**HEALTHY Food Systems**	HAVE YOU...<br>☐ Supported and provided space to grow food (agriculture or neighborhood gardens)?<br>☐ Supported access to healthy and local food?<br>☐ Supported the integration of all of the pieces of the food system in your municipality to enhance the environmental, economic, social, and nutritional health of the community (Producing, Processing, Distributing, Accessing Food, Eating, and Managing Waste)?
**HEALTHY Neighborhood Design**	HAVE YOU...<br>☐ Encouraged mixed land-use designations in your community?<br>☐ Adequately distributed trails, parks or recreation facilities in your community?<br>☐ Promoted or designed your community where people can easily connect with each other and a variety of day-to-day services?<br>☐ Encouraged the Three Cs of healthy neighborhoods: Complete, Compact and Connected?<br>☐ Examined hazard, safety and vandalism concerns in your community
**HEALTHY Natural Environments**	HAVE YOU...<br>☐ Made parks and natural areas of your community accessible to all demographics, safe and connected?

For peer review only

	☐ Protected and enhanced natural areas from development?<br>☐ Incorporated trees and natural features into all new and re-developments, including road work?<br>☐ Incorporated trees and natural features to reduce UV exposure, air pollution, water pollution and urban heat islands?
**HEALTHY Housing**	HAVE YOU...<br>☐ Promoted affordable housing programs?<br>☐ Regulated and enforced housing quality standards?<br>☐ Addressed any high frequencies of tenant/resident relocation or turnover?<br>☐ Designated housing within close proximity to other services and away from environmental hazards?

For peer review only

Supplement Material 3. Round-shaped timetables

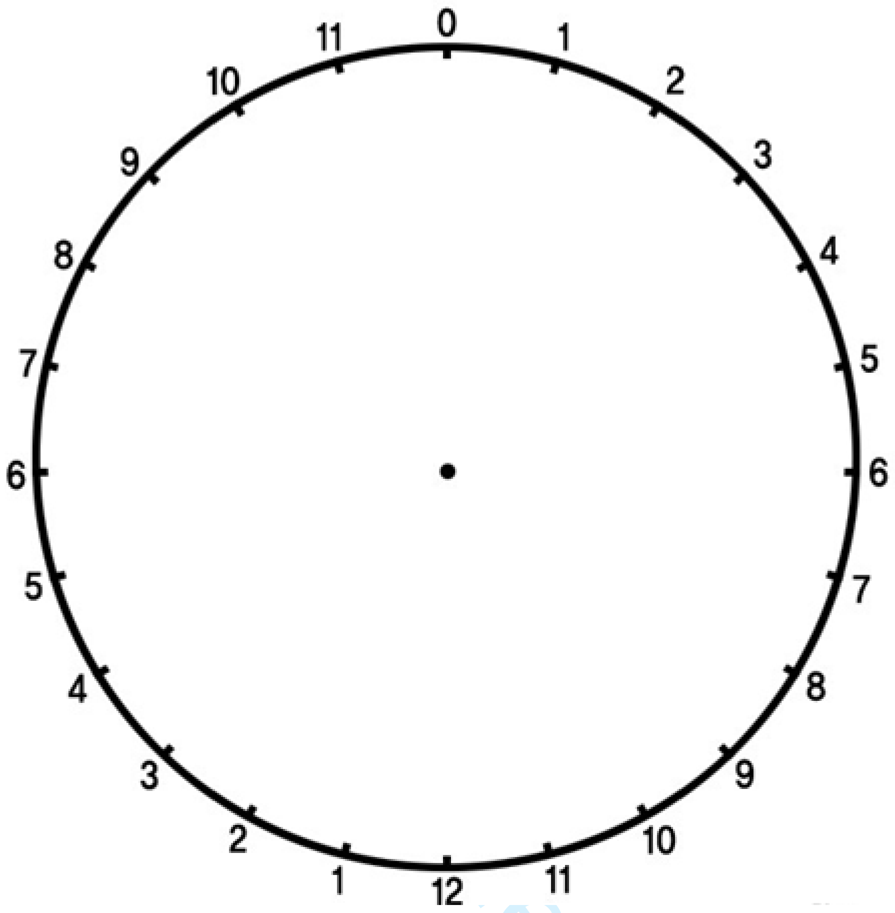

Supplement Material 4. Standards for reporting qualitative research (SRQR) checklist

No.	Topic	Items	Page/line no(s).
**Title and Abstract**			
S1	Title	Concise description of the nature and topic of the study Identifying the study as qualitative or indicating the approach (e.g., ethnography, grounded theory) or data collection methods (e.g., interview, focus group) is recommended	Page 1. L 1-2
S2	Abstract	Summary of key elements of the study using the abstract format of the intended publication; typically includes background, purpose, methods, results, and conclusions	Page 1. L4-28
**Introduction**			
S3	Problem formulation	Description and significance of the problem/phenomenon studied; review of relevant theory and empirical work; problem statement	Page 2. L41-85
S4	Purpose or research question	Purpose of the study and specific objectives or questions	Page 4. L86-96
**Methods**			
S5	Qualitative approach and research paradigm	Qualitative approach (e.g., ethnography, grounded theory, case study, phenomenology, narrative research) and guiding theory if appropriate; identifying the research paradigm (e.g., postpositivist, constructivist/ interpretivist) is also recommended; rationale	Page 4. L99-115
S6	Researcher characteristics and reflexivity	Researchers' characteristics that may influence the research, including personal attributes, qualifications/experience, relationship with participants, assumptions, and/or presuppositions; potential or actual interaction between researchers' characteristics and the research questions, approach, methods, results, and/or transferability	Page 9. L238-243
S7	Context	Setting/site and salient contextual factors; rationale	Page 5. L117-128
S8	Sampling strategy	How and why research participants, documents, or events were selected; criteria for deciding when no further sampling was necessary (e.g., sampling saturation); rationale	Page 5. L130-147
S9	Ethical issues pertaining to human subjects	Documentation of approval by an appropriate ethics review board and participant consent, or explanation for lack thereof; other confidentiality and data security issues	Page 10. L.263-274
S10	Data collection method	Types of data collected; details of data collection procedures including (as appropriate) start and stop dates of data collection and analysis, iterative process, triangulation of sources/methods, and modification of procedures in response to evolving study findings; rationale	Page 6. L148-212
S11	Data collection instruments and technologies	Description of instruments (e.g., interview guides, questionnaires) and devices (e.g., audio recorders) used for data collection; if/how the instrument(s) changed over the course of the study	Page 7. L165-167<br>Page 7. L180-184<br>Page 8. L196-202<br>Page 8. L204-207

For peer review only

S12	Units of study	Number and relevant characteristics of participants, documents, or events included in the study; level of participation (could be reported in results)	Page 5. L131-137
S13	Data processing	Methods for processing data prior to and during analysis, including transcription, data entry, data management and security, verification of data integrity, data coding, and anonymization/de-identification of excerpts	Page 7. L174-176<br>Page 9. L228-232
S14	Data analysis	Process by which inferences, themes, etc., were identified and developed, including the researchers involved in data analysis; usually references a specific paradigm or approach; rationale	Page 9. L213-237
S15	Techniques to enhance trustworthiness	Techniques to enhance trustworthiness and credibility of data analysis (e.g., member checking, audit trail, triangulation); rationale	Page 10. L244-252
**Results/findings**			
S16	Synthesis and interpretation	Main findings (e.g., interpretations, inferences, and themes); might include development of a theory or model, or integration with prior research or theory	N/A*
S17	Links to empirical data	Evidence (e.g., quotes, field notes, text excerpts, photographs) to substantiate analytic findings	N/A*
**Discussion**			
S18	Integration with prior work, implications, transferability, and contribution(s) to the field	Short summary of main findings; explanation of how findings and conclusions connect to, support, elaborate on, or challenge conclusions of earlier scholarship; discussion of scope of application/generalizability; identification of unique contribution(s) to scholarship in a discipline or field	N/A*
S19	Limitations	Trustworthiness and limitations of findings	Page 2. L29-39
**Others**			
S20	Conflict of interest	Potential sources of influence or perceived influence on study conduct and conclusions; how these were managed	Page 11. L281
S21	Funding	Sources of funding and other support; role of funders in data collection, interpretation, and reporting	Page 11. L279-280

N/A: These items do not apply to this manuscript, which is a research protocol.

For peer review only

BMJ Open

Neighborhood environments for a healthy lifestyle among young single-person households experiencing housing poverty in Seoul, South Korea: A spatiotemporal qualitative study protocol

Journal:	*BMJ Open*
Manuscript ID	bmjopen-2023-077234.R2
Article Type:	Protocol
Date Submitted by the Author:	22-Dec-2023
Complete List of Authors:	Kim, Dong Ha; Seoul National University, Institute of Health and Environment<br>Lee, Jihyun; Seoul National University, Department of Public Health Sciences, Graduate School of Public Health<br>Yoo, Seunghyun; Seoul National University, Department of Public Health Sciences, Graduate School of Public Health; Seoul National University, Institute of Health and Environment
**Primary Subject Heading**:	Qualitative research
Secondary Subject Heading:	Public health, Research methods
Keywords:	Health Equity, PUBLIC HEALTH, QUALITATIVE RESEARCH, Quality of Life

SCHOLARONE™
Manuscripts

Neighborhood environments for a healthy lifestyle among young single-person households experiencing housing poverty in Seoul, South Korea: A spatiotemporal qualitative study protocol

ABSTRACT

Introduction

The number of single-person households is increasing globally—including in South Korea, where they account for over 30% of all households. Young single-person households in South Korea face health problems and housing challenges. Both the perceived and objective aspects of the neighborhood environment, as a community asset, play a significant role in sustaining a healthy lifestyle. This study aims to explore and describe the meaning, roles, and spatiotemporal characteristics of neighborhood environments for a healthy lifestyle in young single-person households experiencing housing poverty in Seoul, South Korea.

Methods and analysis

This ongoing study uses an extended qualitative geographic information systems (GIS) approach to explore a district in the city of Seoul that has the highest population density of young single-person households experiencing housing poverty. The study sample comprises young single-person households aged 19–39 years who are experiencing housing poverty in the study area, with an expected saturation point of approximately 55 participants. We employ online and offline recruitment strategies to ensure the inclusion of diverse perspectives and a multimethod approach that combines descriptive and spatiotemporal data collection techniques (e.g., individual in-depth interviews, field observations, and mobile global positioning system (GPS) tracking). The data analysis encompasses thematic and content analyses to understand the neighborhood environment's perceived attributes and the spatiotemporal characteristics of healthy lifestyles. In the integrated analysis, we plan to combine the qualitative findings with living space and daily-life patterns using qualitative software and a hybrid relational database.

Ethics and dissemination

The Institutional Review Board of Seoul National University approved the research protocol on May 18, 2021. The findings will be shared at international conferences and published in academic journals. Additionally, an online seminar will be conducted to share the results with policymakers, researchers, community organizations,

For peer review only

For peer review only - http://bmjopen.bmj.com/site/about/guidelines.xhtml

and health workers working with young single-person households experiencing housing poverty.

Strengths and limitations of this study

- This study provides valuable insights by exploring the lived experiences of young single-person households experiencing housing poverty.
- This study helps identify the meanings and values attached to young single-person households experiencing housing poverty in various places and to understand how these meanings are formed and interpreted.
- This study may be time-consuming, labor-intensive, and costly due to the need to collect and analyze data from multiple sources and devices.
- This study requires a high level of competence and expertise in managing data quality to address the complexities of integrating diverse data sources and ensure the active participation of the target population.
- The interpretation of the study results should consider the characteristics and context of both the study area and participants, particularly in relation to housing poverty.

INTRODUCTION

Single-person households are becoming increasingly common worldwide. In European Union member states, the proportion of single-person households increased from 31% in 2010 to 34% in 2017 (1). According to the United States Census Bureau (2), the proportion of single-person households in the United States increased from 16.7% in 1969 to 28.4% in 2019, and the rate of increase is expected to accelerate over the next decade. Similar to most developed countries, South Korea (hereafter, Korea) has experienced notable household fragmentation in recent years, leading to a trend toward individualized life courses. In 2000, single-person households accounted for only 15.5% of all households in Korea, but had the highest household composition ratio (27.2%) and overtook multi-person households in 2015. This figure had risen even further to 33.4% by 2021 (3).

Compared to the other demographic groups in Korea, young single-person households face a higher risk of health and housing in 2020, 63.2% of young single-person households aged 19–29 were either obese or overweight, 27.7% showed symptoms of depression, and 29.3% were smokers (4). These figures were statistically significantly

For peer review only - http://bmjopen.bmj.com/site/about/guidelines.xhtml

higher than those for multi-person households (5). In 2021, the Korea Research Institute for Human Settlements (6) reported that 60% of young single-person households spent more than 30% of their monthly income on housing rent, which is than five times higher than that of young couple households and households with young adults living with parents. Additionally, according to the 2020 Korea Housing Survey (7), 9.6% of single-person households living in houses with less than 14 m^2 of residential space experienced difficulties in soundproofing, ventilation, heating, and kitchen facilities. These housing conditions are lacking compared to the international minimum housing standards that provide specific facilities and layouts such as kitchens, bathrooms, privacy protections, appropriate hot water supply temperatures, and 25 m^2 of space per person (8). This suggests that young single-person households in Korea may not have sufficient resources within their homes to manage and protect their health adequately. Given the challenges faced by these households, namely, insufficient home resources to adequately address health needs, highlighting the health-promoting factors of the neighborhood environment could be a pivotal strategy for improving the quality of life of those living in single-person households.

Recognizing the unique challenges faced by young, single-person households underscores the urgency of prioritizing and cultivating a healthy lifestyle. A healthy lifestyle, intricately intertwined with the surrounding environment, is a holistic concept that transcends physical health, encompassing mental health, nutrition, and overall quality of life (9). The combination of factors such as regular exercise, a balanced diet, mental well-being, and positive social interactions not only contributes to individual well-being but also interfaces with the neighborhood environment (9). This symbiotic relationship operates synergistically within the holistic framework of a healthy lifestyle. Recognizing the interconnectedness of these elements is critical, as together they form the foundation for wellness and fortification against potential health risks, with the neighborhood environment playing a pivotal role in shaping and supporting these health-promoting behaviors (10).

Neighborhood environment refers to the physical, social, economic, and cultural factors that surround and influence residents within a specific geographic area (11). These factors include land use patterns, street safety, public transportation systems, living facilities, parks and green spaces, housing quality, and social and cultural opportunities. Additionally, the neighborhood environment has perceived and objective aspects (12). The *perceived aspect* of a neighborhood environment refers to how individuals perceive and experience their neighborhood (13). In contrast, the *objective aspect* of the neighborhood environment refers to the physical and

For peer review only - http://bmjopen.bmj.com/site/about/guidelines.xhtml

structural characteristics of the neighborhood that can be measured quantitatively (13). The perceived and objective aspects of the neighborhood environment are significant in shaping individuals' experiences and health outcomes. Perceived aspects influence how individuals behave and engage with their communities, whereas objective aspects affect access to resources and services, safety, and overall well-being (14). By understanding the perceived and objective aspects of the neighborhood environment, policymakers, researchers, and community members can collaborate to create healthier and more livable neighborhoods.

Collecting data on various aspects, such as time, space, and experience, is crucial for comprehensively understanding the perceived and objective neighborhood environments related to healthy lifestyles. The time available for utilizing the neighborhood environment can shape patterns can form a pattern of participation in health behaviors (15), and spatial access to neighborhood environments can affect an individual's ability to maintain a healthy lifestyle (16). Perceptions of neighborhood environments can also affect intentions, practices, and attitudes toward health behaviors (17). Therefore, considering these aspects in data collection can provide a more comprehensive understanding of the relationship between the neighborhood environment and healthy lifestyle of young single-person households experiencing housing poverty.

This study aims to explore and describe the meaning, roles, and spatiotemporal characteristics of neighborhood environments in order to secure healthy lifestyles for young single-person households experiencing housing poverty in Seoul, Korea. The specific research questions are as follows:

1) What perspectives do young single-person households experiencing housing poverty have on their homes and neighborhoods in terms of what would promote a healthy lifestyle?

2) How do young single-person households experiencing housing poverty maintain or attempt to pursue a healthy lifestyle in their neighborhoods?

3) What positive attributes and characteristics of neighborhood environments are necessary to support healthy lifestyles in young single-person households experiencing housing poverty?

4) What is the context and scope of perceived neighborhood boundaries for young single-person households experiencing housing poverty?

METHODS AND ANALYSIS

For peer review only - http://bmjopen.bmj.com/site/about/guidelines.xhtml

Study design

This study employs an extended qualitative GIS (QGIS) research design by adding time-use data from daily life. The original QGIS methodology integrates qualitative and geospatial methods to investigate the rich contextual details of spatial experiences in various formats (18). By merging qualitative methods with GIS-based techniques, QGIS provides an analytical framework for narrative inquiries into spatial experiences that have previously been unfeasible (19, 20). One advantage of visualizing textual information on a map (e.g., perceptions, attitudes, emotions, beliefs, and behaviors) is the ability to explore spatial and environmental contexts that may not be fully elucidated through interviews alone (21). By incorporating time aspects into these benefits, we expect to explore the daily time usage patterns of young single-person households experiencing housing poverty in their neighborhoods.

Figure 1 shows a schematic diagram of the extended QGIS used in this study. This diagram demonstrates how qualitative, spatial, and temporal data can be combined through simultaneous processing to produce a rich contextual understanding of people's lived experiences in their neighborhoods. This distinctive aspect sets our study's strategy apart from a mixed methods approach (22) as it has a unique sequence and arrangement for collecting both qualitative and quantitative data. Additionally, we take a more qualitative-oriented approach compared to traditional QGIS by interpreting GIS information based on patterns rather than concentrating solely on size, frequency, and density.

[Figure 1]

Setting

Our study is currently being conducted in an administrative district with the highest density of young single-person households experiencing housing poverty, Gwanak-gu, which is located in southern Seoul (Figure 2). The district has three administrative neighborhoods with a total area of 29.6 km^2, 60% of which is occupied by mountainous terrain (23). As of September 2021, there were 164,000 single-person households in the district, making it the region with the largest number of single-person households in Korea (23). The main housing type in the district is the multiple complex house with a semi-basement, accounting for 55% of the total houses (23). The average per capita housing area was 28.2 m^2 (approximately 303 ft^2), and the monthly rent for a studio apartment was about 400,000 won (approximately 299.00 USD) in 2021. Gwanak-gu is known for its poor and

For peer review only - http://bmjopen.bmj.com/site/about/guidelines.xhtml

low-cost residential environment (23). In addition, the district has a low-income community ("Go-si-chon" in Korean) where a large number of unemployed young people who prepare for the civil service examinations live in close quarters (23).

[Figure 2]

Study sample and recruitment

Three administrative neighborhoods in Gwanak-gu, where young single-person households are concentrated, are included in our research as study areas. We are currently in the planning phase of conducting stratified purposive sampling (24) for single-person households (aged 19–39) experiencing housing poverty within the study areas. In this study, housing poverty is defined as residing in housing that either 1) fails to meet the minimum housing standards in Korea, or 2) requires more than 30% of and individual's monthly household income (25). The exclusion criteria are individuals who have lived in the study areas for less than six months and those who do not possess electronic and digital devices capable of conducting online interviews. Online and offline recruitment strategies are currently being employed to ensure the inclusion of diverse perspectives from participants of various genders, ages, employment statuses, housing types, and residence periods. Online recruitment involves posting recruitment documents on social network service (SNS) platforms commonly used by young people in the study areas with the cooperation of online community managers. Offline recruitment entails attaching recruitment documents to telephone poles, bulletin boards, and walls located in areas frequented by local young people, as well as visiting public service centers that rent spaces to local young people. Participants will be recruited until data saturation is achieved; that is, when new information no longer produces significant changes to the code book (26). The saturation point of study participants is expected to be approximately 9–17 people per neighborhood, based on previous research (27), and with additional participants to confirm saturation, the number of study participants is expected to be approximately 55.

Data collection

We are currently employing a multi-method approach to gather descriptive and spatiotemporal information on the neighborhood environment to determine what would be conducive for healthy lifestyles among young single-person households experiencing housing poverty. This approach combines descriptive data collection techniques, such as individual in-depth interviews, spatial data collection techniques (e.g., cognitive mapping,

For peer review only - http://bmjopen.bmj.com/site/about/guidelines.xhtml

field observations, and mobile GPS tracking), and temporal data collection techniques (e.g., drawing round-shaped timetables, which are hourly schedules represented in a circular format). The data collection process involves both non-human research (field observations) and human subject research. We are conducting human subject research in the order of individual in-depth interviews using cognitive maps and round-shaped timetables, followed by mobile GPS tracking (Figure 3). Data collection has been ongoing since September 2022.

[Figure 3]

Descriptive data collection

Individual in-depth interviews are an effective and proactive qualitative research method that can be used to understand the lives and experiences of research participants in their language and perspective and to discover their inner world (28). Different types of individual in-depth interviews can be conducted depending on the means, number of research participants, interview format, and openness to interview questions. The most well-known types include open, semi-structured, and structured interviews. For this ongoing study, we are conducting semi-structured interviews with young single-person households experiencing housing poverty. The interviews are being conducted face-to-face and through the online videoconferencing platform Zoom, depending on participants' preferences. The researchers first explain the study and then obtain consent from individuals regarding their willingness to participate. Additionally, they confirm their consent to record and use their interviews, which will be transcribed and used only for the purpose of analysis. Participants are informed that they can stop the interview at any time if they wish, and the informed consent form makes it clear that participation is entirely voluntary.

After obtaining consent, we conduct a survey to collect demographic information, including sex, age, occupation, length of residence, housing characteristics, and health status. Following the survey, we conduct one-on-one in-depth interviews using semi-structured questions to gain insights into the perceived attributes of neighborhood environments related to healthy lifestyles (Supplement Material 1). We record and transcribe the interviews into electronic documents with an average turnaround time of five days. The expected average interview time per participant is approximately one hour and 30 minutes.

Spatial data collection

Field observation is a spatial research method in which a researcher observes phenomena related to a research

For peer review only - http://bmjopen.bmj.com/site/about/guidelines.xhtml

topic while moving around a research site (29). Currently, we are following a procedure for conducting field observations. We use Google Maps and Street View to examine the neighborhood environment and plan observation paths, schedules, and boundaries. To investigate the physical characteristics of the neighborhood environment and the contents that change depending on the observation time, we use The Healthy Community Checklist (30). We are manually recording the collected information on field observation sheets (Supplement Material 2), which will later be digitized for future reference and organized by date and neighborhood.

Techniques to visually represent an individual or group's mental model of the physical environment are known as cognitive mapping (31), which involves asking participants to either map from their memories or annotate existing maps with information that reflects their perceptions, knowledge, and experiences of the environment. In this study, the researchers conduct activities during one-on-one interviews with the participants to create cognitive maps. Participants are asked to mark the map with reference to places and experiences related to healthy living. Upon completion of the cognitive map, the researchers ask the participants to explain the main activities in the marked places, their reason for selecting them, and any difficulties they encountered while using them. Finally, participants are asked to indicate the environmental characteristics of their neighborhood (within what they perceive to be its boundaries) that impact their health. We expect cognitive maps to provide insights into how people navigate and use their environments, perceive and prioritize different features and landmarks, and conceptualize their spatial relationships.

Mobile GPS can easily track the spatial movement of participants' daily activity radius in real-time (32). In this study, the participants wear the Garmin Forerunner device and record their daily lives during the survey period. The mobile GPS devices help confirm the range of participants' walking activity radii and identify their movement paths and the locations where they stayed in the neighborhood. The participants' data, including distance traveled, duration of movement, movement path, and place of residence are automatically synchronized with Google Maps and stored daily. Based on previous research evidence the research team conducts the tracking once for a period of three days, including two weekdays and one day on the weekend (33).

Temporal data collection

During the one-on-one in-depth interviews, we use a technique that creates a round-shaped timetable to understand participants' 24-hour daily routines, which are separated into weekdays and weekends. We divide a 24-hour day into equal segments and assign activities or events to each segment based on the start and end times.

For peer review only

For peer review only - http://bmjopen.bmj.com/site/about/guidelines.xhtml

Each segment is labeled and represented by a circle, similar to a clock face (Supplemental Material 3). We ask participants to complete a daily schedule based on weekday and weekend routines. Once completed, the researchers review participants' schedules and ask for additional explanations of their daily neighborhood activities. Finally, we ask participants to describe activities related to a healthy lifestyle and explain why, when, and how often they engaged in them. We expect the round timetable to outline how individuals allocate their time, the duration and frequency of different activities, and the temporal patterns of their behavior.

Data analysis and integration

We will conduct six stages of thematic analysis (34) for the in-depth interview data. In the first stage, we will develop a general framework for participants' perceptions of the neighborhood environment related to a healthy lifestyle by repeatedly reading the interview data. In the second stage, we will conduct an open coding process to identify all meaning units related to the perception of the neighborhood environment, which affects participants' healthy lifestyles, such as meaning, attributes, and roles. In the third stage, we will compare and contrast the relationships between the generated codes to identify categories. In the fourth stage, we will review the tentatively derived themes to determine whether they match the research purpose and reflect the data content. In the fifth stage, we will refine the derived themes into precise definitions through a recursive process of returning data for comparative analysis. Based on the final analysis results, we will provide a detailed description and present appropriate explanations and discussions on the research purpose.

We plan to perform content analysis using spatiotemporal data. Content analysis offers a systematic and objective approach to draw accurate inferences from verbal, visual, or written data, thus enabling the description and quantification of specific phenomena (35). We will extract information, including time usage patterns (amount of time and time of day), activity types, and activity range (within/outside the neighborhood), from the timetables created by individual study participants and analyze this information through overlapping analysis. We will convert the cognitive map data provided by participants and the field observation data surveyed by the researchers into a digital map using the Quantum GIS program in order to quantitatively analyze spatial characteristics (location, density, radius, distance, and distribution). We will analyze the mobile log data collected through mobile GPS tracking for neighborhood mobility patterns using the statistical software R and Google Maps.

During the integrated analysis phase, we plan to combine the qualitative results with living space and daily

For peer review only - http://bmjopen.bmj.com/site/about/guidelines.xhtml

life pattern results using Atlas.ti qualitative software and a hybrid relational database. The hybrid relational database will allow us to create a descriptive data database under the attribute table of spatiotemporal data, which can integrate various types of qualitative data, such as images, texts, and videos, into spatial data (36). Atlas.ti can process text data and non-text multimedia data and be linked to GIS and codes (37).

Researcher characteristics and reflexivity

The research team consists of individuals aged 30 and above who are majoring in public health sciences at the university in the study area. This composition enhances the team's understanding of the neighborhoods and facilitates participant recruitment. However, it is worth noting that none of the researchers belong to the demographic of young adults living in housing poverty, thus potentially introducing bias or leading to an overestimation in reading and interpreting the context of these young adults' lives.

Trustworthiness

We will apply Lincoln and Guba's (1985) criteria (38) for rigor in qualitative research (credibility, transferability, dependability, and confirmability) to enhance the trustworthiness of this study. To enhance the credibility of our research, we plan to cross-verify the perspectives, experiences, and behaviors of individuals collected from various sources using multiple qualitative methods. To improve transferability, we will provide a detailed description of the phenomenon, enabling a comparison with background data to establish the study's context. We will offer an in-depth methodological description of the study, which promotes replicability and enhances dependability. Additionally, the study will be audited by external experts with many years of experience in qualitative research to enhance confirmability.

Reporting of findings

The findings of this qualitative study will be reported in accordance with the Standards for Reporting Qualitative Research (SRQR) statement (39). The application of SRQR is not confined to the writing phase alone; these standards can aid researchers in planning qualitative studies and in meticulously documenting processes and decisions made throughout the study (Supplementary Material 4).

Patient and public involvement

There is no patient and public involvement in the design, conduct, reporting, or dissemination plans of this

For peer review only - http://bmjopen.bmj.com/site/about/guidelines.xhtml

study. However, we obtain advice from key informants in the study area, including public service providers and long-term residents, to ensure pathways for participant recruitment and to enhance the research team's understanding of the local context.

ETHICS AND DISSEMINATION

The study was approved by the Institutional Review Board of Seoul National University on May 18, 2021 (IRB number 2106/002-018). Before participating in the study, participants are asked to provide written informed consent to ensure complete anonymity and confidentiality. The informed consent form explains in detail the purpose of the recording, possible risks and discomfort, potential benefits, confidentiality of information, and withdrawal procedures. Participants' anonymity is protected, and no identifiable characteristics are specified in the recorded conversations. We will disseminate the findings to the academic society by presenting them at international conferences on urban health and publishing them in academic journals. In addition, we plan to conduct an online seminar/webinar to share and discuss our research findings with policymakers, researchers, community organizations, and health workers who plan and implement policies and programs for young single-person households experiencing housing poverty.

Contributors D.H.K., J.L., and S.Y. conceived the study. D.H.K., J.L., and S.Y. contributed to the development of the study design and final protocols for sample selection and data collection. D.H.K. and J.L. contributed to writing the manuscript. S.Y. provided critical editing and approved the final version of the paper.

Funding This work was supported by a National Research Foundation (NRF) grant funded by the Korean government (Ministry of Science, ICT, and Future Planning; NRF- 2020R1A2C2012463).

Competing interests None declared.

Patient consent for publication Not required.

Provenance and peer review Not commissioned; externally peer reviewed.

REFERENCES

For peer review only - http://bmjopen.bmj.com/site/about/guidelines.xhtml

1 Eurostat. Eurostat Regional Yearbook: 2019 Edition. Eurostat Publications Office 2019. doi: 10.2785/1522

2 United States Census Bureau. One-person households on the rise [online]. 2019. https://www.census.gov/library/visualizations/2019/comm/one-person-households.html (accessed 7 April 2023).

3 Statistics Korea. Single-Person Households in 2021 Statistics (Korean). Statistics Korea 2021.

4 Ministry of Health and Welfare, Korea Disease Control and Prevention Agency. Announcement of the Results of the 8th National Health and Nutrition Survey for the 2nd Year. 2020.

5 Shin, MA. Comparative study on health behavior and mental health between one person and multi-person households: Analysis of data from the National Health and Nutrition Examination Surveys (Korean). KSW 2019;14(4):11-23.

6 Park M, Woo J. Complementary strategies for housing vulnerability by age group of single-person households. Sejong: Korea Research Institute for Human Settlements 2021:16–18.

7 Korea Research Institute for Human Settlements. 2020 Housing Survey. Sejong: Ministry of Land, Infrastructure and Transport 2020:91–92.

8 Yoo D, Lee J, Jang E. An international comparative study to shift from minimum to decent housing standard (Korean). JAIK 2022;38:47–58.

9 Korp P. The symbolic power of 'healthy lifestyles'. Health Sociol Rev 2008;17(1):18-26.

10 Font JC, Hernández-Quevedo C, McDonald JT, Variyam JN. Understanding healthy lifestyles: The role of choice and the environment. Appl Econ Perspect Policy 2013;1-6.

11 Ding D, Sallis JF, Kerr J, et al. Neighborhood environment and physical activity among youth: A review. Am J Prev Med 2011;41:442–55. doi: 10.1016/j.amepre.2011.06.036.

12 Orstad SL, McDonough MH, Stapleton S, et al. A systematic review of agreement between perceived and objective neighborhood environment measures and associations with physical activity outcomes. Environ Behav 2017;49:904–932.

13 Kim DH, Yoo S. How does the built environment in compact metropolitan cities affect health? A systematic review of Korean studies. Int J Environ Res Public Health 2019;16(16):2921. doi: 10.3390/ijerph16162921

14 Ma L, Dill J. Associations between the objective and perceived built environment and bicycling for

For peer review only - http://bmjopen.bmj.com/site/about/guidelines.xhtml

transportation. J Transp Health 2015;2(2):248–255.

15 Yamaguchi M, Praditsorn P, Purnamasari SD, et al. Measures of perceived neighborhood food environments and dietary habits: A systematic review of methods and associations. Nutrients 2022;14(9):1788.

16 Kang CD. The S+ 5Ds: spatial access to pedestrian environments and walking in Seoul, Korea. Cities 2018;77:130–141.

17 Sawitri DR, Hadiyanto H, Hadi SP. Pro-environmental behavior from a social-cognitive theory perspective. Procedia Environ Sci 2015;23:27–33.

18 Cope M, Elwood S. Qualitative GIS: A mixed methods approach. London: SAGE Publications 2009:1–12.

19 Mennis J, Mason MJ, Cao Y. Qualitative GIS and the visualization of narrative activity space data. Int J Geogr Inf Sci 2013;27(2):267–291.

20 Kim J, Kim DH, Lee J, et al. A scoping review of qualitative geographic information systems in studies addressing health issues. Soc Sci Med 2022;314:115472.

21 Yoo S, Kim DH. Perceived urban neighborhood environment for physical activity of older adults in Seoul, Korea: A multimethod qualitative study. Prev Med 2017;103:S90–S98.

22 Creswell, JW. A concise introduction to mixed methods research. Thousand Oaks, CA: Sage Publications 2014:34-50.

23 Gwanak-gu office. General status of Gwanak-gu [online]. 2022. https://www.gwanak.go.kr/site/gwanak/11/11101010000002016051207.jsp (accessed 15 December 2022).

24 Patton MQ. Qualitative research and evaluation methods (3rd ed.). Thousand Oaks, CA: Sage 2002:240.

25 Park SY. Housing poverty in Korea (Korean). Health and Welfare Policy Forum 2012;2012(2):33-46. doi: 10.23062/2012.02.5.

26 Guest G, Bunce A, Johnson L. How many interviews are enough? An experiment with data saturation and variability. Field Methods 2006;18(1): 59–82.

27 Hennink M, Kaiser BN. Sample sizes for saturation in qualitative research: A systematic review of empirical tests. Soc Sci Med 2022;292:114523.

28 Legard R, Keegan J, Ward K. In-depth interviews. In: Qualitative Research Practice: A Guide for Social Science Students and Researchers. London: SAGE Publications 2003:138–169.

29 Mulhall A. In the field: Notes on observation in qualitative research. J Adv Nurs 2003;41(3):306–313.

For peer review only - http://bmjopen.bmj.com/site/about/guidelines.xhtml

30 Lambton Public Health. Built Environment Healthy Community Checklist [online]. 2017. https://lambtonpublichealth.ca/resource/built-environment-healthy-community-checklist-2017/ (accessed 31 October 2022).

31 Moeser SD. Cognitive mapping in a complex building. Environ Behav 1988;20(1):21–49.

32 Jeong, GS, Kong SH. GIS-based advanced positioning technique for mobile GPS (Korean). JKICS 2015;40 (11):2261–2270.

33 Zenk SN, Matthews SA, Kraft AN, Jones KK. How many days of global positioning system (GPS) monitoring do you need to measure activity space environments in health research? Health Place 2018;51:52–60.

34 Braun V, Clarke V. Conceptual and design thinking for thematic analysis. Qual Psychol 2022;9(1): 3-6.

35 Downe-Wamboldt, B. Content analysis: method, applications, and issues. Health Care Women Int 1992;13 (3):313–321.

36 Jung JK, Elwood S. Extending the qualitative capabilities of GIS: Computer-aided qualitative GIS. Trans GIS 2010;14(1):63–87.

37 Smit B. Introduction to ATLAS.ti for mixed analysis. In: The Routledge Reviewer's Guide to Mixed Methods Analysis. New York: Routledge 2021:331–342.

38 Lincoln YS, Guba EG. Establishing trustworthiness. Naturalistic Inquiry 1985;289(331):289-327.

39 O'Brien BC, Harris IB, Beckman TJ, Reed DA, Cook DA. Standards for reporting qualitative research: A synthesis of recommendations. Acad Med 2014;89(9):1245-1251.

only

For peer review only - http://bmjopen.bmj.com/site/about/guidelines.xhtml

369 **FIGURE CAPTIONS**

370 **FIGURE 1 Schematic diagram of this study**

371 **FIGURE 2 Study site**

372 **FIGURE 3 Data collection process**

For peer review only

For peer review only - http://bmjopen.bmj.com/site/about/guidelines.xhtml

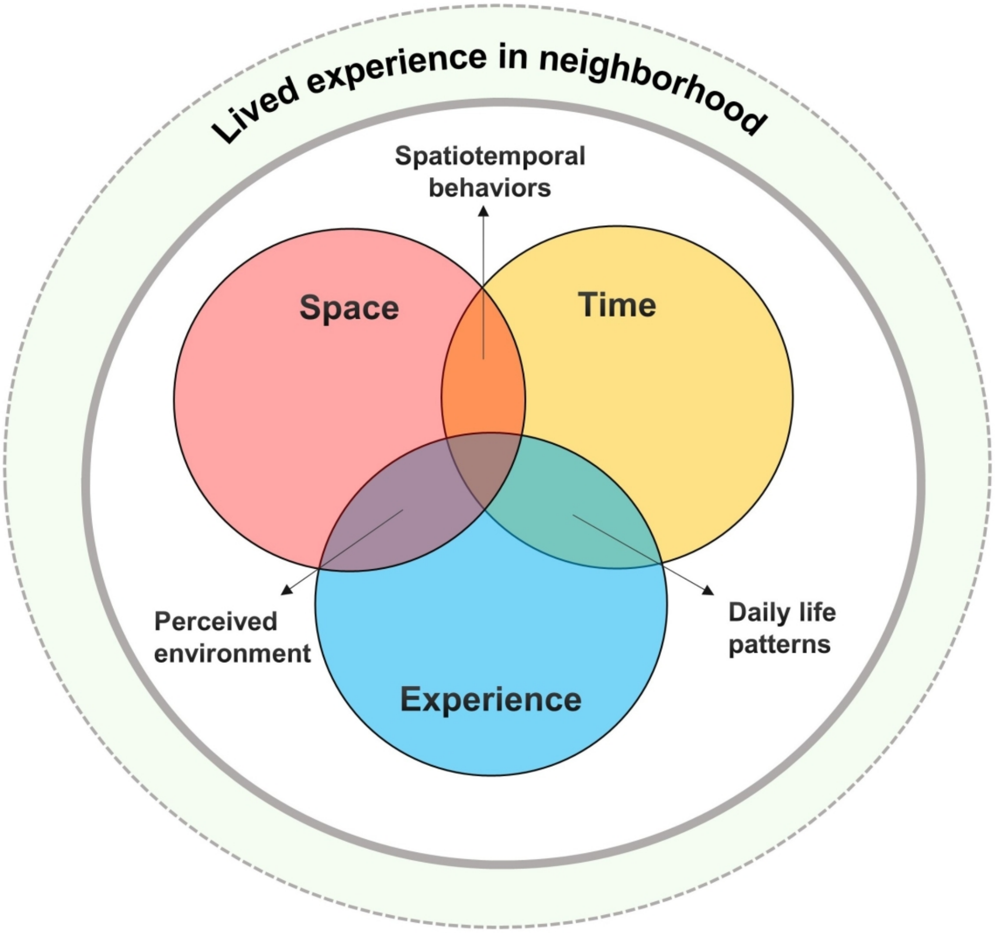

FIGURE 1. Schematic diagram of this study

185x173mm (300 x 300 DPI)

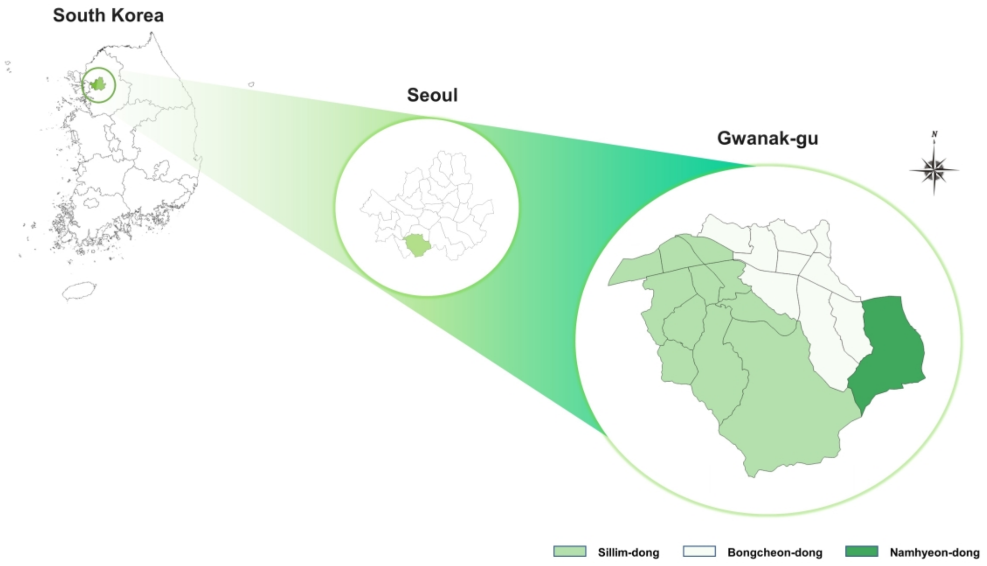

FIGURE 2. Study site

338x190mm (300 x 300 DPI)

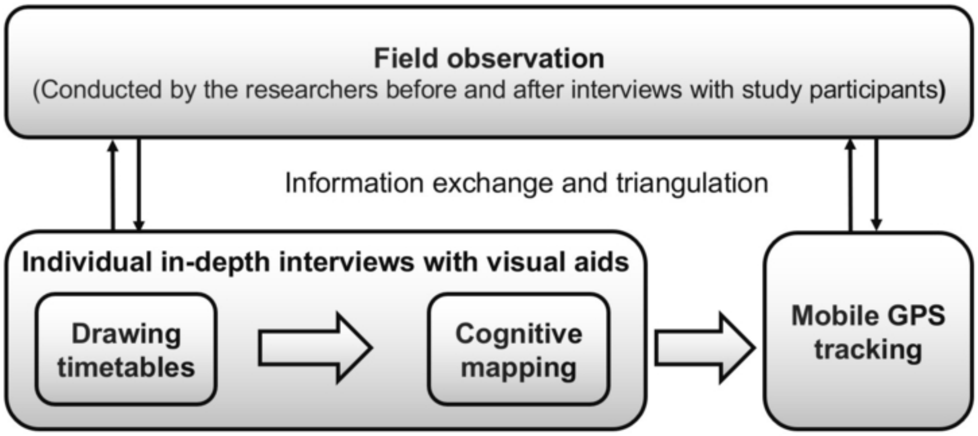

Phase	Who	What (Research activities)	When		
			Before the Interview	During the interview	After the interview
1	Researcher	Field observation			
2	Researcher & Participant	In-depth interview			
		Drawing timetables			
		Cognitive mapping			
3	Participant	Mobile GPS tracking			

FIGURE 3. Data collection process

62x47mm (300 x 300 DPI)

Supplement Material 1. Interview questions

Activities	Interview questions
Prepare a timetable (Confirmation by displaying on the timetable based on participant responses)	**1. We'd like to draw a timeline of your daily life. Describe a typical day in your life.**<br>- How long has this lifestyle been going on?<br>- Is there a reason for this change in lifestyle (if it's different before or after COVID-19)?<br><br>**2. We'd like to ask your thoughts on "healthy living".**<br>- What do you think "healthy living" is about practicing or enjoying in your daily life?<br>- As you reflect on your day, describe any activities you maintain or try to do to stay healthy.<br>- If there's something you'd like to try to do for your health and you're not able to, please explain what you can't do and why.<br>- Thinking about your day, describe any activities that you do that contribute to poor health.<br>- Describe any reasons why you engage in the activity despite its health risks.
Prepare a neighborhood map (Confirm by displaying on a map based on participant responses)	**3. We would like to ask you about places, spaces that you associate with 'healthy living'.**<br>- Looking at the map, please explain where you do/would like to do each of the activities related to "healthy living" that you mentioned earlier.<br>- Where is this space/place located? Please mark it on the map.<br>- What activities do you typically do in that space/location?<br>- Why did you choose that space/location, if any?<br>- What are the challenges of utilizing that space/location?<br>(*Need to check: the environment of your current home)
	**4. We'd like to ask you about your neighborhood's health-related environment.**<br>- Fill in the blanks in the sentence "One thing about my neighborhood, [ ], affects my health" and be specific about why.<br>- Show me on a map where you think my neighborhood exemplifies the characteristics you just described.<br>- Plot the route from your home to the location on a map.<br>- If you've been to or used these places, please describe what you

For peer review only

	did there, for what purpose, and what it was like.<br>- If there's one thing you'd like to see improved in your neighborhood for healthy living, please describe it.<br>- Assuming you live in the neighborhood you live in now for a long time to come, how do you think the meaning of your neighborhood or your use of it will change?<br>- Based on what we've talked about so far, plot on a map where you think your "neighborhood" is and where it ends.

For peer review only

Supplement Material 2. Checklist of field observation (Lambton Public Health, 2017)

Check areas	Check points
**HEALTHY Transportation Networks**	HAVE YOU...<br>☐ Supported a variety of transportation modes that are convenient and safe for all ages?<br>☐ Encouraged retail and commercial spaces that support active transportation with connections to cycling paths, trails, sidewalks or transit, if applicable?<br>☐ Required or encouraged homes and places of employment to be within a comfortable walking or biking distance of frequent destinations such as schools, parks, shops, and transit stops, if applicable?<br>☐ Designed roadways and access to buildings and services as pedestrian friendly
**HEALTHY Food Systems**	HAVE YOU...<br>☐ Supported and provided space to grow food (agriculture or neighborhood gardens)?<br>☐ Supported access to healthy and local food?<br>☐ Supported the integration of all of the pieces of the food system in your municipality to enhance the environmental, economic, social, and nutritional health of the community (Producing, Processing, Distributing, Accessing Food, Eating, and Managing Waste)?
**HEALTHY Neighborhood Design**	HAVE YOU...<br>☐ Encouraged mixed land-use designations in your community?<br>☐ Adequately distributed trails, parks or recreation facilities in your community?<br>☐ Promoted or designed your community where people can easily connect with each other and a variety of day-to-day services?<br>☐ Encouraged the Three Cs of healthy neighborhoods: Complete, Compact and Connected?<br>☐ Examined hazard, safety and vandalism concerns in your community
**HEALTHY Natural Environments**	HAVE YOU...<br>☐ Made parks and natural areas of your community accessible to all demographics, safe and connected?

For peer review only

	☐ Protected and enhanced natural areas from development?<br>☐ Incorporated trees and natural features into all new and re-developments, including road work?<br>☐ Incorporated trees and natural features to reduce UV exposure, air pollution, water pollution and urban heat islands?
**HEALTHY Housing**	HAVE YOU...<br>☐ Promoted affordable housing programs?<br>☐ Regulated and enforced housing quality standards?<br>☐ Addressed any high frequencies of tenant/resident relocation or turnover?<br>☐ Designated housing within close proximity to other services and away from environmental hazards?

For peer review only

Supplement Material 3. Round-shaped timetables

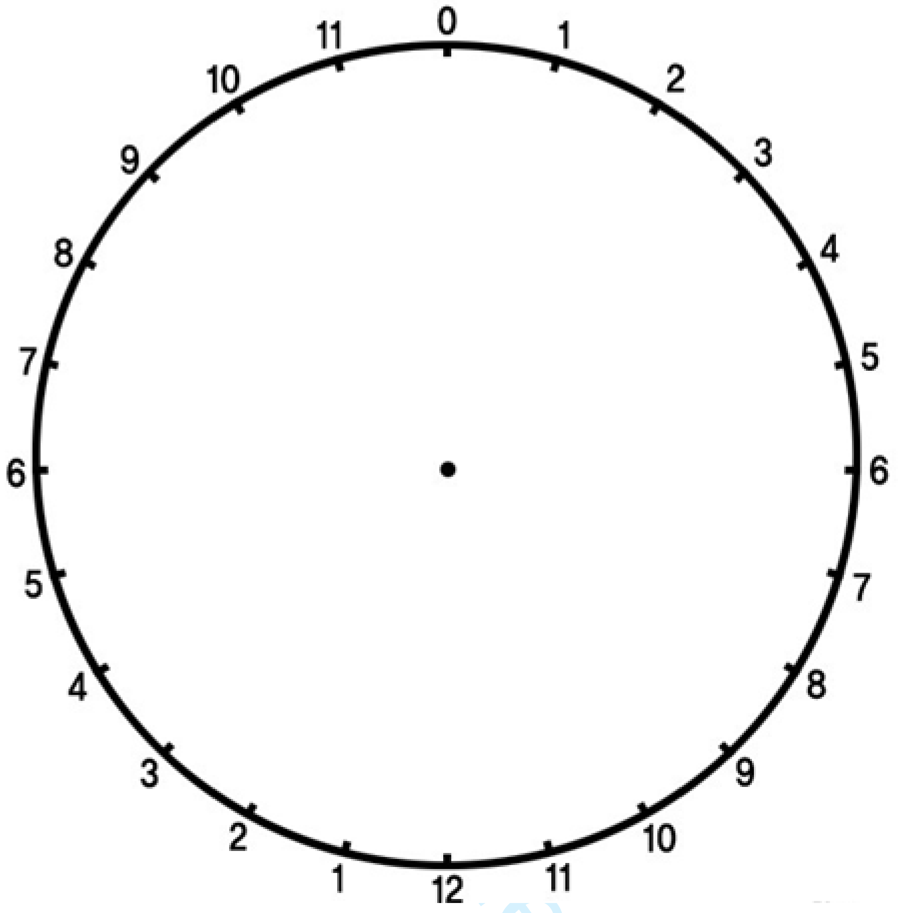

ew only

Supplement Material 4. Standards for reporting qualitative research (SRQR) checklist

No.	Topic	Items	Page/line no(s).
**Title and Abstract**			
S1	Title	Concise description of the nature and topic of the study Identifying the study as qualitative or indicating the approach (e.g., ethnography, grounded theory) or data collection methods (e.g., interview, focus group) is recommended	Page 1. L 1-2
S2	Abstract	Summary of key elements of the study using the abstract format of the intended publication; typically includes background, purpose, methods, results, and conclusions	Page 1. L4-28
**Introduction**			
S3	Problem formulation	Description and significance of the problem/phenomenon studied; review of relevant theory and empirical work; problem statement	Page 2. L41-85
S4	Purpose or research question	Purpose of the study and specific objectives or questions	Page 4. L86-96
**Methods**			
S5	Qualitative approach and research paradigm	Qualitative approach (e.g., ethnography, grounded theory, case study, phenomenology, narrative research) and guiding theory if appropriate; identifying the research paradigm (e.g., postpositivist, constructivist/ interpretivist) is also recommended; rationale	Page 4. L99-115
S6	Researcher characteristics and reflexivity	Researchers' characteristics that may influence the research, including personal attributes, qualifications/experience, relationship with participants, assumptions, and/or presuppositions; potential or actual interaction between researchers' characteristics and the research questions, approach, methods, results, and/or transferability	Page 9. L238-243
S7	Context	Setting/site and salient contextual factors; rationale	Page 5. L117-128
S8	Sampling strategy	How and why research participants, documents, or events were selected; criteria for deciding when no further sampling was necessary (e.g., sampling saturation); rationale	Page 5. L130-147
S9	Ethical issues pertaining to human subjects	Documentation of approval by an appropriate ethics review board and participant consent, or explanation for lack thereof; other confidentiality and data security issues	Page 10. L.263-274
S10	Data collection method	Types of data collected; details of data collection procedures including (as appropriate) start and stop dates of data collection and analysis, iterative process, triangulation of sources/methods, and modification of procedures in response to evolving study findings; rationale	Page 6. L148-212
S11	Data collection instruments and technologies	Description of instruments (e.g., interview guides, questionnaires) and devices (e.g., audio recorders) used for data collection; if/how the instrument(s) changed over the course of the study	Page 7. L165-167<br>Page 7. L180-184<br>Page 8. L196-202<br>Page 8. L204-207

For peer review only

S12	Units of study	Number and relevant characteristics of participants, documents, or events included in the study; level of participation (could be reported in results)	Page 5. L131-137
S13	Data processing	Methods for processing data prior to and during analysis, including transcription, data entry, data management and security, verification of data integrity, data coding, and anonymization/de-identification of excerpts	Page 7. L174-176<br>Page 9. L228-232
S14	Data analysis	Process by which inferences, themes, etc., were identified and developed, including the researchers involved in data analysis; usually references a specific paradigm or approach; rationale	Page 9. L213-237
S15	Techniques to enhance trustworthiness	Techniques to enhance trustworthiness and credibility of data analysis (e.g., member checking, audit trail, triangulation); rationale	Page 10. L244-252
**Results/findings**			
S16	Synthesis and interpretation	Main findings (e.g., interpretations, inferences, and themes); might include development of a theory or model, or integration with prior research or theory	N/A*
S17	Links to empirical data	Evidence (e.g., quotes, field notes, text excerpts, photographs) to substantiate analytic findings	N/A*
**Discussion**			
S18	Integration with prior work, implications, transferability, and contribution(s) to the field	Short summary of main findings; explanation of how findings and conclusions connect to, support, elaborate on, or challenge conclusions of earlier scholarship; discussion of scope of application/generalizability; identification of unique contribution(s) to scholarship in a discipline or field	N/A*
S19	Limitations	Trustworthiness and limitations of findings	Page 2. L29-39
**Others**			
S20	Conflict of interest	Potential sources of influence or perceived influence on study conduct and conclusions; how these were managed	Page 11. L281
S21	Funding	Sources of funding and other support; role of funders in data collection, interpretation, and reporting	Page 11. L279-280

N/A: These items do not apply to this manuscript, which is a research protocol.

For peer review only
